# Quantitative videodensitometric assessment of aortic regurgitation in Myval, Sapien, and Evolut THV series: Results from the LANDMARK trial

**DOI:** 10.1016/j.ijcha.2025.101804

**Published:** 2025-09-29

**Authors:** Elfatih A. Hasabo, Niels van Royen, Ignacio J Amat-Santos, Martin Hudec, Matjaz Bunc, Alexander IJsselmuiden, Peep Laanmets, Daniel Unic, Bela Merkely, Renicus S Hermanides, Vlasis Ninios, Marcin Protasiewicz, Benno J W M Rensing, Pedro L Martin, Fausto Feres, Manuel De Sousa Almeida, Eric van Belle, Axel Linke, Alfonso Ielasi, Matteo Montorfano, Mark Webster, Konstantinos Toutouzas, Emmanuel Teiger, Francesco Bedogni, Michiel Voskuil, Manuel Pan, Oskar Angerås, Won-Keun Kim, Jürgen Rothe, Ivica Kristić, Vicente Peral, Ben J.L. Van den Branden, Ashokkumar Thakkar, Udita Chandra, Cagri Ayhan, Dina Neiroukh, Mahmoud Y. Nosir, Magdi S. Yacoub, Sanaa Ali, Mohamad Altamimi, Hesham Elzomor, Patrick W Serruys, Andreas Baumbach, Osama Soliman

**Affiliations:** a Royal College of Surgeons in Ireland (RCSI) University of Medicine and Health Sciences, Dublin, Ireland; b Cardiovascular Research Institute Dublin (CVRI), Mater Private Network, Eccles Street, Dublin D07 KWR1, Ireland; c Department of Cardiology, Radboud University Hospital, Nijmegen, Netherlands; d Centro de Investigación Biomédica en red - Enfermedades Cardiovasculares (CIBERCV), Instutito de salud Carlos III, Madrid, Spain; e Department of Cardiology, Hospital Clinico Universitario de Valladolid, Valladolid, Spain; f Department of Acute Cardiology, Middle-Slovak Institute of Cardiovascular Diseases, Banska Bystrica, Slovakia; g Department of Cardiology, University Medical Centre Ljubljana, Ljubljana, Slovenia; h Department of Cardiology, Amphia Hospital, Breda, Netherlands; i Department of Interventional Cardiology, Maastricht University Medical Center, Maastricht, Netherlands; j Zuyderland Hospital, Limburg, Netherlands; k Department of Invasive Cardiology, North Estonia Medical Centre, Tallinn, Estonia; l Department of Cardiac and Transplant Surgery, University Hospital Dubrava, Zagreb, Croatia; m Heart and Vascular Centre, Semmelweis University Heart and Vascular Center, Budapest, Hungary; n Department of Cardiology, Isala Hospital, Zwolle, the Netherlands; o Department of Cardiology, European Interbalkan Medical Center, Thessaloniki, Greece; p Department of Cardiology, Institute of Heart Diseases, Wroclaw Medical University, Wroclaw, Poland; q Department of Cardiology, St Antonius Hospital, Nieuwegein, the Netherlands; r Department of Interventional Cardiology, University Hospital of Gran Canaria Dr Negrín, Las Palmas, Spain; s Department of Invasive Cardiology, Instituto Dante Pazzanese de Cardiologia, Sao Paulo, Brazil; t CHRC, NOVA Medical School, NOVA University Lisbon, Lisbon, Portugal; u Department of Interventional Cardiology, Lille University Hospital, Lille, France; v Department of Internal Medicine and Cardiology, University Clinic, Heart Center Dresden, University of Technology Dresden, Dresden, Germany; w Department of Interventional Cardiology, IRCCS Ospedale Galeazzi Sant’Ambrogio, Milan, Italy; x School of Medicine, Vita-Salute San Raffaele University, Milan, Italy; y Interventional Cardiology Unit IRCCS San Raffaele Scientific Institute, Milan, Italy; z Department of Cardiology, Auckland City Hospital, Auckland, New Zealand; aa Department of Cardiology, Hippokration Hospital, Athens, Greece; ab Department of Interventional Cardiology, Henri-Mondor University Hospital, Creteil, France; ac Department of Cardiology, IRCCS Policlinico San Donato, San Donato Milanese, Milan, Italy; ad Department of Cardiology, University Medical Center Utrecht, Utrecht, Netherlands; ae Department of Cardiology, University Hospital Reina Sofía, University of Córdoba, IMIBIC, CIBERCV, Córdoba, Spain; af Department of Cardiology, Sahlgrenska University Hospital, Gothenburg, Sweden; ag Department of Molecular and Clinical Medicine, Institute of Medicine, University of Gothenburg, Gothenburg, Sweden; ah Department of Cardiology & Angiology, University of Giessen and Marburg, Giessen, Germany; ai Department of Cardiology, Kerckhoff Heart Center, Bad Nauheim, Germany; aj Department of Cardiology and Angiology, Campus Bad Krozingen, University Heart Center-University of Freiburg, Germany; ak Faculty of Medicine, University of Freiburg, Germany; al Department of Cardiology, University Hospital of Split, Split, Croatia; am Department of Cardiology University Hospital Son Espases, Health Research Institute of the Balearic Islands (IdISBa), Palma, Balearic Islands, Spain; an Department of Clinical Research, Meril Life Sciences Pvt. Ltd., Vapi, India; ao Discipline of Cardiology, Saolta Healthcare Group, Galway University Hospital, Health Service Executive, H91 YR71 Galway, Ireland; ap Discipline of Medicine, School of Medicine, University of Galway, Galway, Ireland; aq Centre for Cardiovascular Medicine and Devices, William Harvey Research Institute, Queen Mary University of London and Barts Heart Centre, London, UK; ar Cleveland Clinic, London, UK

**Keywords:** Videodensitometry, Regurgitant fraction, Transcatheter aortic valve implantation, Aortic regurgitation, Balloon post-dilatation

## Abstract

**Background:**

The quantitative videodensitometric aortography (QVDA) has reliably quantified post-TAVI aortic regurgitation (AR). However, this method has not yet been evaluated in randomized trials comparing various transcatheter heart valve (THV) systems. Here, we investigated the QVDA of AR following TAVI for severe aortic stenosis among Myval, Sapien, and Evolut THV series as part of the LANDMARK trial.

**Methods:**

The final aortograms, either without or after balloon post-dilatation (BPD) were analyzed using the advanced CAAS-A-Valve 2.1.2 software. The regurgitant fraction (RF) was computed and categorized into none/trace AR (RF < 86 %), mild AR (6 % ≤ 8RF ≤ 817 %), and moderate/severe AR (RF > 17 %).

**Results:**

Five hundred ninety-six final analyzable aortograms and 97 aortograms following BPD were included in the analysis. The BPD resulted in a significant reduction of RF in the Myval [12.0(6.0–18.5) vs 2.0(1.0, 5.5);p = 0.0002], Sapien[18.0(1.0–19.0) vs. 2.0(1.0–3.0); p = 0.04206] and Evolut [10.5 (6.0–15.0) vs 5.0 (1.0–8.0); p = 0.0009]. The rate of final RF > 17 % was lower in the Myval(2.0 %) compared to Evolut(8.00 %), but similar to the Sapien series (4.0 %)(P_Myval-Sapien_ = 0.2333, P_Myval-Evolut_ = 0.0057). In the as-treated population, the Myval series demonstrated a comparable RF to the Sapien series, but a significantly lower RF compared to the Evolut [Myval: 3.0 %(1.0–7.0), Sapien:3.0 %(1.0–7.0), Evolut:5.0 %(1.0–10.0)], P_Myval-Sapien_ = 0.8997,P_Myval-Evolut_ = 0.0010].

**Conclusion:**

The QVDA highlights the superior performance of the Myval THV series compared to the Evolut THV series, with the lowest rate of moderate/severe RF among the three THV series, and could be used with echocardiography to help in detecting cases with none/trace AR.

## Graphical abstract

Central illustration: Quantitative videodensitometric assessment of final aortogram post-TAVI.

**Figure:**
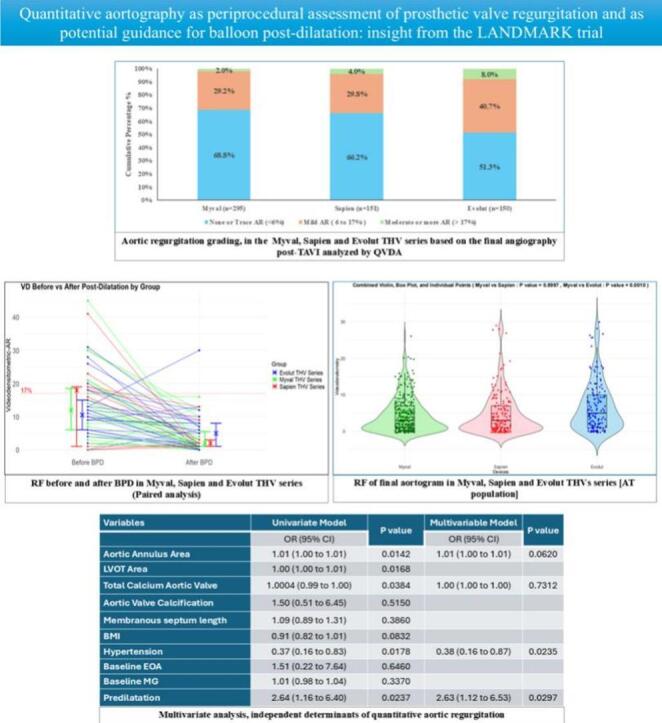

## 1 Introduction

Following transcatheter aortic valve implantation (TAVI) for severe aortic stenosis, prosthetic valve may exhibit paravalvular leak (PVL), varying in severity from trivial to severe aortic regurgitation (AR), potentially affecting the durability of the valve and the longer-term outcome following the procedure [1,2].

In its early phase of development, TAVI showed a relatively high incidence of moderate/severe PVL, whereas with the evolving technology and the latest generation of transcatheter heart valves (THV), the percentage of moderate and severe PVL and prosthetic valve regurgitation has considerably declined when compared with the early generation of THVs [[3], [4], [5], [6]].

Several imaging modalities have been used to assess and adjudicate the severity of PVL, and echocardiography is currently the most used and recommended modality for assessing PVL following TAVI [7]. However, the use of transthoracic echocardiography (TTE), transesophageal echocardiography (TEE) and the presence of an echocardiographist in the catheterization laboratory are nowadays uncommon and the diagnosis of PVL is mostly made retrospectively, outside the cath lab without the option to amend the regurgitation periprocedurally. Imaging modalities other than echocardiography may be used for PVL assessment, including visual aortography, quantitative videodensitometric aortography (QVDA), and cardiac magnetic resonance [8,9]. Only the first two modalities −visual aortography and QVDA- can be applied online during the procedure. In contrast with the visual and subjective assessment of AR, the QVDA measures the difference in the time-density curve of the angiographic contrast medium administered in the aortic root area and regurgitated in the left ventricular outflow tract (LVOT) to calculate the regurgitation fraction (RF) either “on line” in the lab or offline for a retrospective evaluation [10,11]. The technique has been validated, found accurate and reproducible in an in-silico mock circulation system [12,13] as well as in clinical studies after TAVI in patients with either aortic stenosis or for AR [[14], [15], [16]]. It has been established that a cut-off point of RF > 17 %, −a threshold level of regurgitation associated with late mortality after TAVI [14,17] and corresponding to a moderate/severe degree of AR as determined by TTE, TEE [12] and magnetic resonance [15,18,19].

Balloon post-dilatation (BPD) is performed in patients with PVL after TAVI mainly for mitigation or elimination of PVL [20]. In the LANDMARK trial, BPD was performed in 118 patients (15.36 %) post-TAVI [3]. Therefore, this post-hoc analysis of the LANDMARK trial aims to provide a detailed QVDA assessment of the final aortogram, including the one following BPD and to attempt to identify the factors associated with the presence of residual PVL among the three valves series.

## 2 Methods

### 2.1 Study population and design

This is a post-hoc analysis of the LANDMARK randomized, open-label trial, which consisted of 768 participants receiving either the Myval, Sapien, or Evolut THVs series.

This multicenter study included patients with severe AS who underwent a post-procedural aortogram in the cath lab for immediate assessment of PVL, whenever the aortogram was quantitatively analyzable. Details of inclusion criteria were published in the LANDMARK trial’s primary publication [3].

### 2.2 Balloon post-dilatation

Supplementary Table 1 shows the details of these reasons as well as the imaging modalities used for the decision of BPD. The detection and reduction in PVL were assessed during the procedure mainly by aortography (75.4 %, Supplementary Table 1). Diverse motivations were involved in the decision-making of the BPD performance, but the most common reason for performing BPD was the presence of PVL and suboptimal valve expansion documented by angiography. The nominal balloon size and its adjusted volumetric filling to appropriately correct the PVL was left at the operator's discretion. The severity of PVL has been adjudicated using Valve Academic Research Consortium 3 (VARC-3) as follows: none or trace, mild, moderate or severe [7] by echocardiography, and QVDA assessment.

### 2.3 Quantitative videodensitometric aortographic assessment of aortic regurgitation

Angiography as guidance during the procedure or as a final assessment of AR was done in the aortic root during TAVI by injecting contrast dye, resulting in a total amount of contrast used for each THV [Median (Q1-Q3) = Myval: 141.0 (100–180) ml; Sapien: 135.0 (101–195.5) ml; Evolut: 134.0 (100–200) ml].

For the AR assessment, the pigtail catheter was placed maximally 2 cm above the edge of the prosthetic metallic frame during the injection of contrast (20–30 ml) for the Myval THV series and Sapien THV series and inside the outflow region of the Evolut THV metallic frame. Final aortograms, following an eventual BPD, were analyzed by a central independent Core lab who was was blinded to the valve type and the outcomes, by anonymizing received cases to the core lab.

CAAS-A Valve 2.1.2 (PieMedical Imaging, The Netherlands) was the software used for the QVDA assessment of AR. The QVDA assessment relies on the ratio of the amount of contrast detected in the aorta versus the amount detected in the outflow tract of the left ventricle displayed as two time-density curves in the two regions of interest (ROI):

1- Aortic root, where the contrast is injected.
2- Left ventricular outflow tract (the obligatory pathway of regurgitation toward the left ventricle) where the regurgitant contrast is detected.

**Fig. 1:**
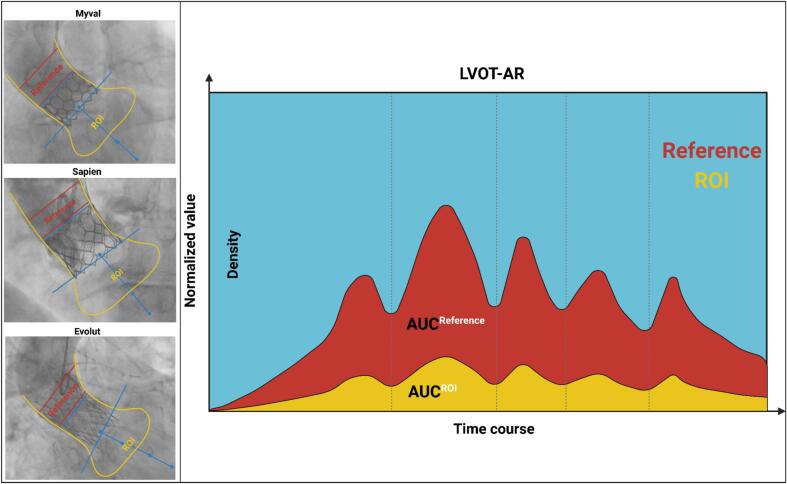
Representation of the quantitative videodensitometric calculation of regurgitant fraction with videodensitometry.

The software measures the ratio difference in density between the left ventricular outflow tract and the aortic root (LVOT/AR). (Fig. 1). It provides the parametric RF (expressed in %) of LVOT-AR on a continuous scale ranging from 0 to 100 %. This technique has been used in previously published clinical studies [14,15,17,18,21]. Moreover, the three categorical cut-off points for RF defining the three levels of regurgitation were established in previous clinical studies and are categorized as follows: none or trace AR (RF < 86 %), mild AR (6 % ≤ 8RF ≤ 17 %), and moderate/severe AR (RF > 17 %) [15,22].

### 2.4 Post-dilatation balloon diameter/annulus diameter ratio

Multislice computed tomography (MSCT) assessment was done by TAVI Core lab (India), and the post-dilatation balloon diameter/annulus diameter ratio was measured as follows: [(post-dilatation balloon diameters– annulus diameter)/ (post-dilatation balloon diameters) × 100].

### 2.5 Statistical analysis

Continuous data were presented as mean ± standard deviation (SD), while RF was presented as median (Interquartile range; IQR), and categorical data was reported in numbers with percentages. Participants were categorized according to the type of THV used. The paired student’s *t*-test was used to compare paired data between the two groups, and the independent student’s *t*-test was used for unpaired comparisons. One-way ANOVA and Chi-square tests were used to compare continuous and categorical variables between the three groups, respectively. Concordant Rate, discordant Rate, sensitivity, specificity, area under the curve (AUC), positive predictive value (PPV), and negative predictive value (NPV) were calculated to find the correlation between QVDA and echocardiography at discharge. Univariate and multivariate logistic regression was used to find the predictors of moderate/severe PVL. A P-value of ≤ 0.05 was considered significant, and the analysis was done using R (version 4.3.3).

## 3 Results

### 3.1 Study population and baseline participants' characteristics and yield of QVDA analyzability

**Fig. 2:**
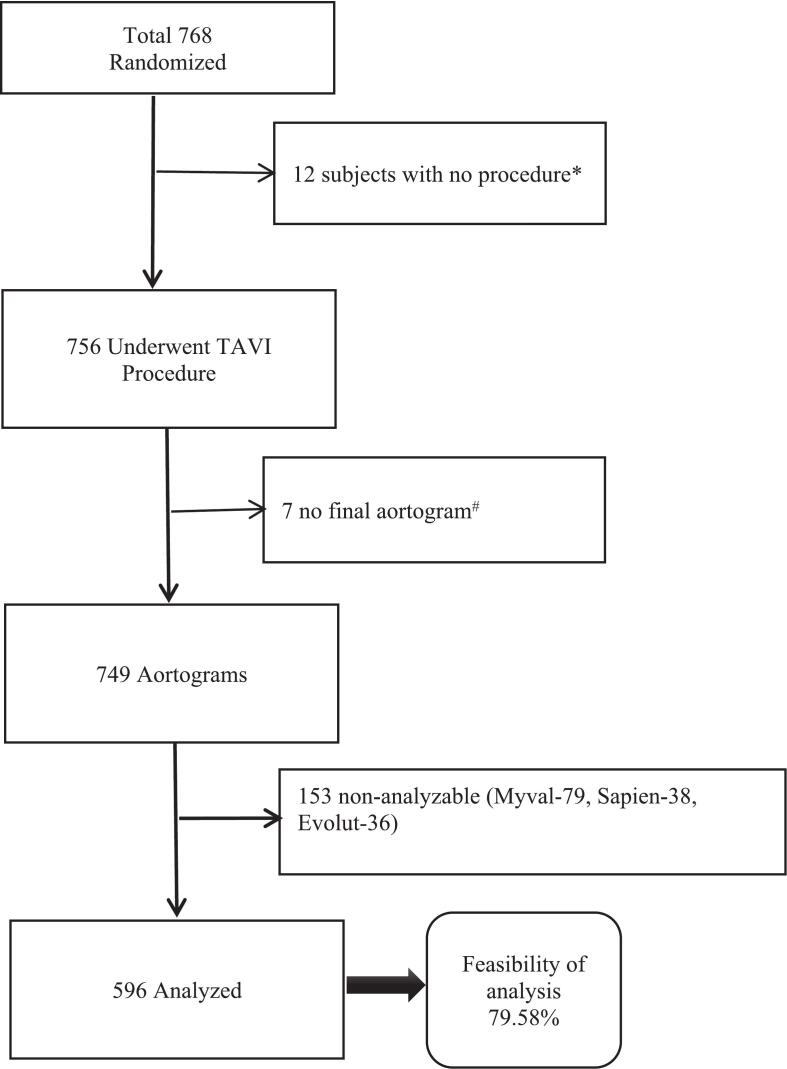
Flow chart diagram of aortogram analysis.

Among the 768 treated participants, 749 (97.5 %) had an aortogram at the end of their TAVI procedures. Of these final aortograms, 596 (79.58 %) were analyzable; the rest were considered non-analyzable for different reasons. The flow diagram of the study and the reasons for the non-analyzability of the aortograms is depicted in Fig. 2 and Supplementary Table 2.

**Fig. 3:**
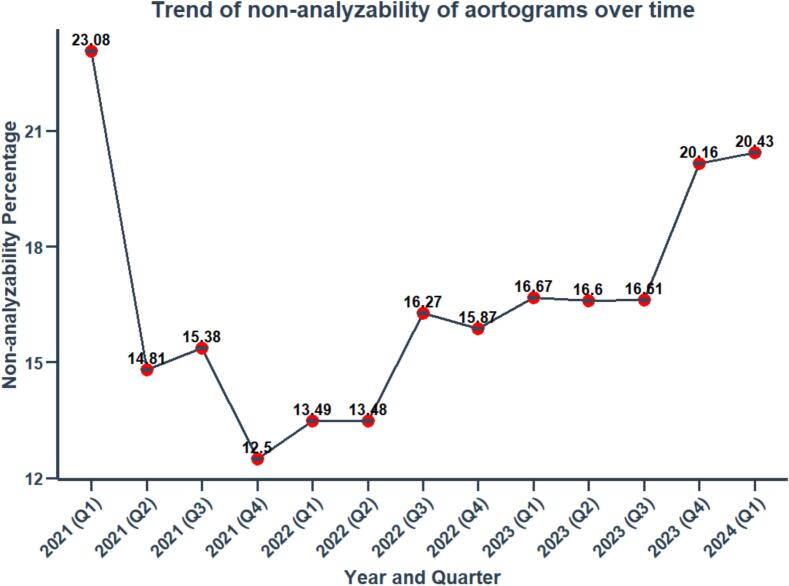
Line chart showing non-analysabilty over time.

On average the analyzability was 79.58 %. The reasons for this suboptimal level of analyzability are tabulated in Supplementary Table 2, and the most common reasons for non-analyzability were breathing motion, coronary arteries shadowing overlapping ROI, and poor image quality, while some of them have multiple causes of non-analyzability. Despite quality control and prospective protocol, monitoring records of the core lab indicate that there was a deterioration of the analyzability over time with increasing heterogeneity of acquisition, especially among the investigators that joined in a late phase of the trial enrolment (Supplementary Table 3 and Fig. 3). It is reassuring that the patients with or without analyzable aortogram had comparable demographic and lesional baselines and were equally distributed in the three arms.

Among them, 295 participants were randomized to the Myval THV series, 151 to the Sapien THV series, and 150 to the Evolut THV series. Baseline demographic and lesional characteristics in the three valves series are tabulated in Table 1, while hemodynamic, procedural and lesional characteristics are presented in Supplementary Table 4. In general, the hemodynamics, clinical and lesional characteristics of non-analyzable final aortograms (n = 153) were similar to those of the participants with analyzable final aortograms. (Supplementary Table 5).

**Table 1:** Baseline characteristic of analyzable aortogram in Myval, Sapien and Evolut THV series.

Baseline Characteristics	Myval THV series (N = 295)	Sapien THV series (N = 151)	Evolut THV series (N = 150)
Age, (year)	80.0 ± 5.9 (n = 295)	81.1 ± 5.2 (n = 151)	79.8 ± 5.2 (n = 150)
Female, (%)	155 (52.5)	65 (43.1)	69 (46.0)
Body mass index (kg/m2)	28.0 ± 4.8 (n = 295)	27.6 ± 4.1 (n = 151)	28.1 ± 5.2 (n = 150)
Body surface area (m2)	1.9 ± 0.2 (n = 295)	1.9 ± 0.2 (n = 151)	1.9 ± 0.2 (n = 150)
Society of Thoracic Surgeons score	3.2 ± 2.6 (n = 295)	3.2 ± 2.1 (n = 151)	3.0 ± 1.8 (n = 150)
Low score (<4)	228 (77.3)	118 (78.2)	116 (77.3)
Intermediate score (4–8)	56 (19.0)	27 (17.9)	32 (21.3)
High score (>8)	11 (3.7)	6 (4.0)	2 (1.3)
EuroSCORE II	3.2 ± 2.1 (n = 42)	2.9 ± 2.6 (n = 30)	7.3 ± 18.7 (n = 18)
New York Heart Association (NYHA)	n = 295		
Class I	12 (4.1)	5 (3.3)	6 (4.0)
Class II	128 (43.4)	67 (44.4)	68 (45.3)
Class III	141 (47.8)	77 (51.0)	66 (44.0)
Class IV	14 (4.8)	2 (1.3)	10 (6.7)
Medical history			
Hypercholesterolaemia	35 (11.9)	1 (0.7)	28 (18.7)
Hypertension	193 (65.4)	98 (64.9)	97 (64.7)
Current smoker	80 (27.1)	28 (18.5)	39 (26.0)
Alcohol consumption	72 (24.4)	17 (11.3)	47 (31.3)
Current Diabetes Mellitus, (%)	82 (27.8)	37 (24.5)	41 (27.3)
Stroke	6 (2.0)	2 (1.3)	5 (3.3)
Atrial fibrillation, (%)	67 (22.7)	38 (25.2)	42 (28.0)
Chronic obstructive pulmonary disease, (%)	27 (9.2)	15 (9.9)	16 (10.7)
Myocardial infarction, (%)	21 (7.1)	9 (5.96)	10 (6.7)
Coronary artery disease, (%)	44 (14.9)	24 (15.9)	22 (14.7)
Prior coronary artery bypass grafting, (%)	10 (3.4)	9 (6.0)	9 (6.0)
Prior percutaneous coronary intervention, (%)	20 (6.8)	6 (4.0)	12 (8.0)
Prior balloon aortic Valvuloplasty, %	4 (1.4)	−	−
Cerebrovascular accident, (%)	3 (1.0)	0 (0.0)	1 (0.67)
Porcelain aorta or hostile chest Procedural Characteristics), (%)	−	−	−
Peripheral vascular disease, (%)	2 (0.7)	2 (1.3)	1 (1.3)
Overall frailty, (%)	42 (14.2)	25 (16.6)	18 (12.0)
Pulmonary hypertension, (%)	8 (2.7)	1 (0.7)	3 (2.0)
Permanent pacemaker, (%)	9 (3.1)	5 (3.3)	8 (5.3)
Left bundle branch block, (%)	6 (2.0)	9 (6.0)	10 (6.7)
Right bundle branch block, (%)	10 (3.4)	9 (6.0)	9 (6.0)
Estimated glomerular filtration rate < 60 mL/min	127/281 (45.2)	67/145 (46.2)	72/142 (50.7)
Estimated glomerular filtration rate < 30 mL/min	39/281 (13.9)	23/145 (15.9)	17/142 (12.0)

### 3.2 Occurrence of BPD and core lab angiographic quantitative assessment of AR post-BPD

A total of 118 participants [Myval: 38 (10 %), Sapien: 19 (10.1 %), and Evolut: 61 (32.5 %)] underwent a BPD. The average post-dilation nominal balloon diameter is 23.7 ± 3.6 mm, and the post-dilatation balloon diameter vs annulus diameters ratio is 1.0 ± 0.1. Ninety-seven post-dilatation aortograms were available. Overall, post-BPD patients had none/trace AR in 57 (58.8 %) patients, mild AR in 35 (36.1 %), and moderate/severe AR in 5 (5.2 %) patients on QVDA. Moreover, after BPD, no patient in the Myval THV series had RF > 17 %, while the Sapien and Evolut THV series reported 5.9 % (n = 1) and 8.5 % (n = 4), respectively. The group-wise data are presented in Supplementary Table 6.

**Fig. 4:**
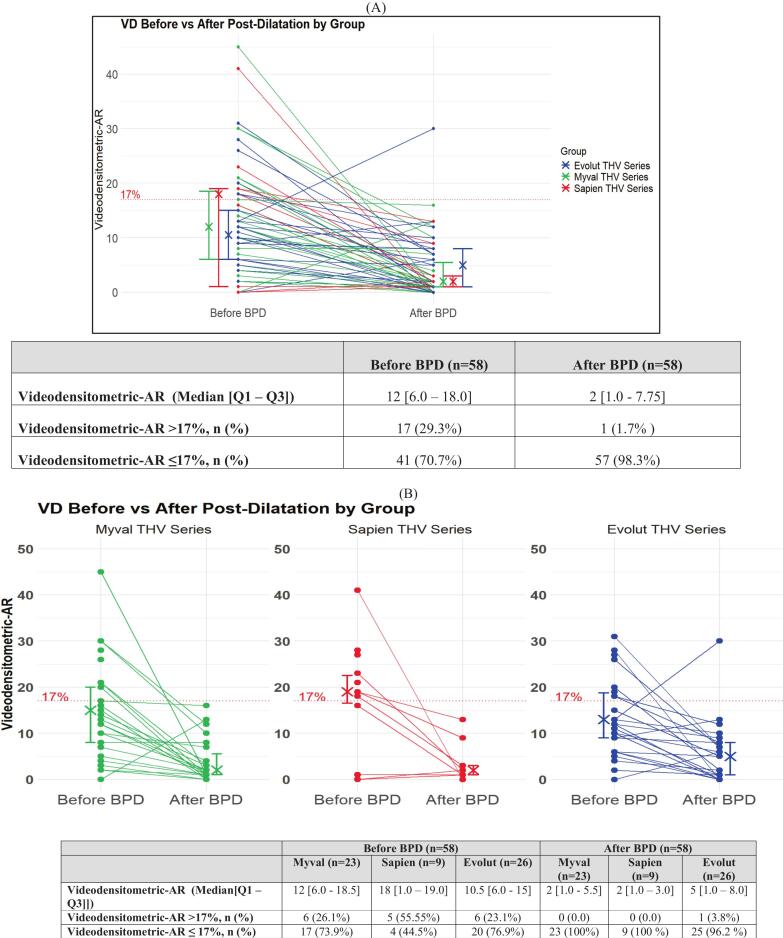
RF before and after BPD in (A) overall participants, (B) Myval, Sapien and Evolut THVs series.

The RF showed significant improvement after BPD in Myval THV series [Median (Q1-Q3): 12.0 (6.0–––18.5) vs 2.0 (1.0, 5.5); p = 0.0002], Sapien THV series [18.0 (1.0 – 19.0) vs. 2.0 (1.0––3.0); p = 0.04206), and Evolut THV series (10.5 (6.0–15.0) vs 5.0 (1.0–8.0); p = 0.0009). The RF’s after BPD didn’t show significant differences across the three THV series [Myval THV series: 2.0 (1.0–5.5) vs. Sapien THV series: 2.0 (1.0–3.0) and Evolut THV series: 5.0 (1.0–8.0); p = 0.6225]. (Fig. 4) (Supplementary Table 6).

### 3.3 Regurgitation fraction of the final aortograms among the three valves

**Fig. 5:**
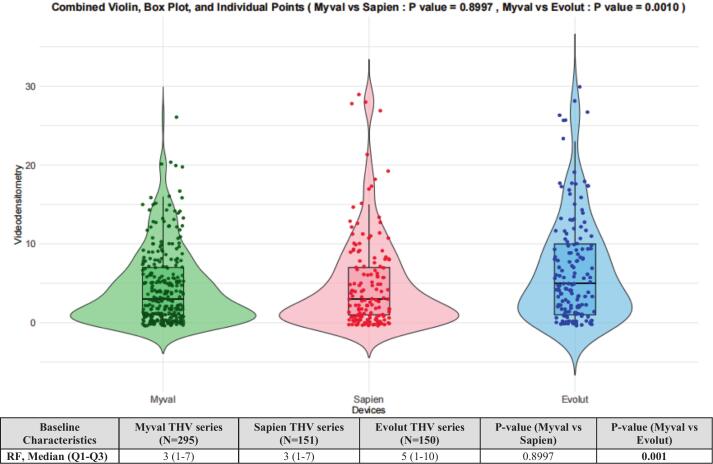
RF of final aortogram in Myval, Sapien and Evolut THVs series.

The QVDA of AR for the final aortogram was expressed in individual values of RF and revealed comparable results for the Myval THV and the Sapien THV series (3.0 [1.0–7.0] vs 3.0 [1.0–7.0], p = 0.8997), but these rates of RF in Myval and Sapien THV series were both smaller than in the Evolut THV series (3.0 [1.0–7.0] vs 5.0 [1.0–10.0], p = 0.001). (Fig. 5).

**Fig. 6:**
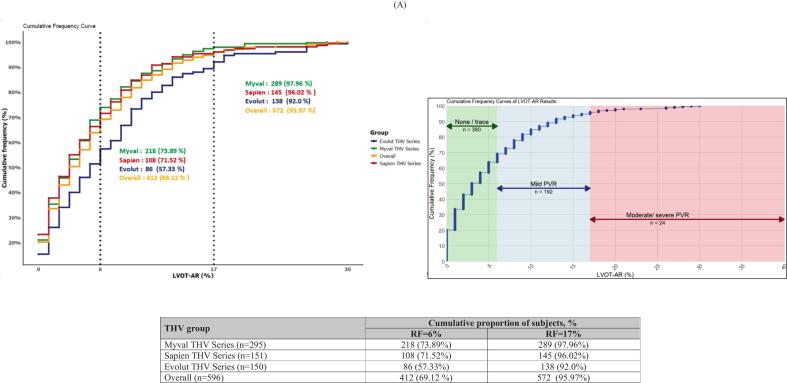

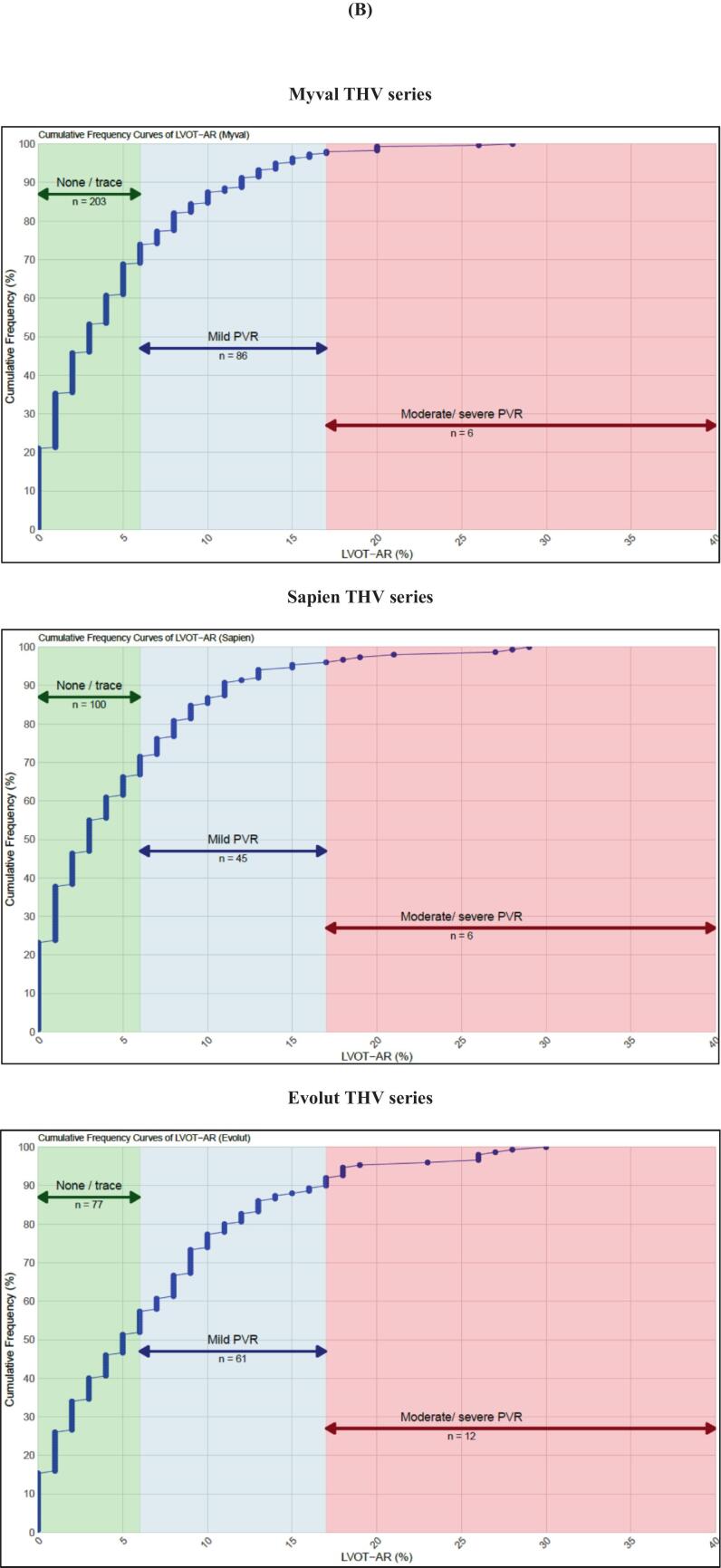
Cumulative incidence of RF in (A) overall, (B) Myval, Sapien and Evolut THV series.

The cumulative frequency curves of RF after TAVI for overall participants and per THVs series are shown in Fig. 6. For RF per device size, intermediate sizes of the Myval THV series showed median (Q1-Q3) RF of 21.5 mm, 24.5 mm, and 27.5 mm were 4.0 (1.0–7.0) %, 3.0 (1.0––7.0) %, and 2.5 (1.0–4.8) %, respectively. RF of size 30.5 mm is reported only once and had RF ≤ 0.17. Sizes 26 mm and 29 mm of Myval reported 2 cases each with RF > 17 and one for size 23 mm. Number of participants with RF > 17 assigned to Sapien THV series were found in size 23 mm (n = 2), 26 mm (n = 3), and 29 mm (n = 2). However, most of the cases in the Evolut THVs with RF > 17 were found in device size 29 mm (n = 8), and further details of videodensitometry per device size are presented in Supplementary Table 7.

### 3.4 Cross correlation of AR assessment by quantitative aortograms post-TAVI and echocardiography at discharge

On the analyzable final aortogram post-TAVI, the severity of AR by QVDA was quantified as a continuous variable and categorized as none/trace AR (n = 380, 63.8 %), mild AR (n = 192, 32.2 %) and moderate/severe AR (n = 24, 4.0 %). (Supplementary Table 5).

**Fig. 7:**
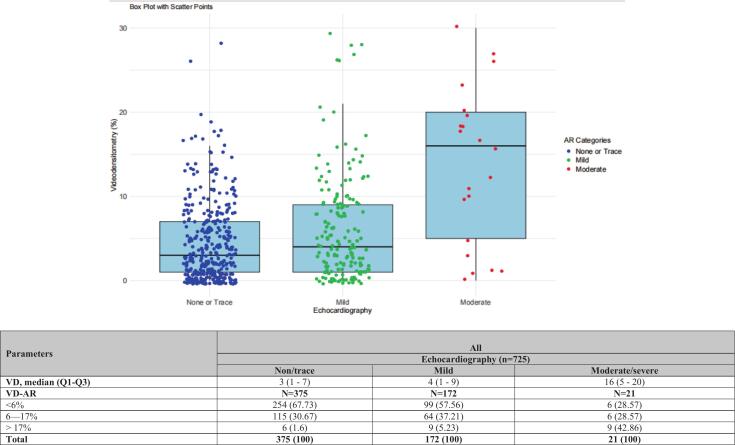
Correlation of Echocardiography at discharge point with videodensitometry.

Aortic regurgitation on echocardiography acquired at discharge in these patients with a final analyzable aortogram post-TAVI (n = 568) showed none/trace AR (n = 375, 66.0 %), mild AR (n = 172, 30.3 %), and moderate/severe AR (n = 21, 3.7 %) (Table 2). Fig. 7 showed the distribution of VD-AR correlated with echocardiography at the discharge visit.

**Table 2:** Correlation of RFpost-TAVI and echocardiography at discharge visit in patients with both analyzable QVDA and echocardiography.

Parameters	All		
	Echocardiography (n = 725)		
	Non/trace	Mild	Moderate/severe
RF, median (Q1-Q3)	3 (1–––7)	4 (1–––9)	16 (5–––20)
RF	N = 375	N = 172	N = 21
<6%	254 (67.73)	99 (57.56)	6 (28.57)
6–17 %	115 (30.67)	64 (37.21)	6 (28.57)
> 17 %	6 (1.6)	9 (5.23)	9 (42.86)
Total	375 (100)	172 (100)	21 (100)

Among the three valves, the Myval THV series showed none/trace AR in 68.8 %, mild AR in 29.2 %, and moderate/severe AR in 2.0 %. The Sapien THV series results of AR were none/trace in 66.2 %, mild in 29.8 %, and moderate/severe in 4.0 %, and the Evolut THV series showed the following findings: none/trace AR (51.3 %), mild AR (40.7 %), and moderate/severe AR (8.0 %). (Supplementary Fig. 1).

The correlation between QVDA and echocardiography in assessing AR showed the following: concordance rate: 29.79 %, discordant rate: 16.93 %, sensitivity: 42.1 %, specificity: 70.8 %, AUC: 0.564, PPV: 45.6 %, NPV: 67.7 %, Kappa: 0.131. The model showed poor predictive performance, while it is somewhat better at predicting true negatives (specificity: 70.8 %, NPV: 67.7 %), it has poor true positive predictive values (sensitivity: 42.1 %, PPV: 45.6 %). The low AUC (0.564) and Kappa (0.131) further emphasize the weak agreement and discrimination.

### 3.5 Multivariate analysis, independent determinants of quantitative aortic regurgitation and probabilistic prediction model

Multivariate analysis identified hypertension (OR: 0.38 [95 % CI: 0.16–––0.87], p = 0.0235) and predilatation (OR: 2.63 [95 % CI: 1.12–––6.53], p = 0.0297) as independent predictors for moderate/severe AR. However, univariate analysis showed aortic annulus area, LVOT area, total aortic calcium, predilatation and hypertension as independent predictors for moderate/severe AR. (Table 3).

**Table 3:** Univariate and multivariate predictors of moderate/severe AR of final aortogram assessed via QVDA.

Variables	Univariate Model	P value	Multivariable Model	P value
	OR (95 % CI)		OR (95 % CI)	
Aortic Annulus Area	1.0067 (1.0014–––1.0121)	0.0142	1.01 (1.00–––1.01)	0.062
LVOT Area	1.0055 (1.0009–––1.0101)	0.0168		
Total Calcium Aortic Valve	1.0005 (1.00–––1.0009)	0.0384	1.00 (1.00–––1.00)	0.7312
Aortic Valve Calcification	1.50 (0.51–––6.45)	0.515		
Membranous septum length	1.09 (0.89–––1.31)	0.386		
BMI	0.91 (0.82–––1.01)	0.0832		
Hypertension	0.37 (0.16–––0.83)	0.0178	0.38 (0.16–––0.87)	0.0235
Baseline EOA	1.51 (0.22–––7.64)	0.646		
Baseline MG	1.01 (0.98–––1.04)	0.337		
Predilatation	2.64 (1.16–––6.40)	0.0237	2.63 (1.12–––6.53)	0.0297
Abbreviation CI: Confidence interval, OR: Odd ratio				

## 4 Discussion

The present post-hoc analysis of the LANDMARK trial provides detailed information about quantitative videodensitometric assessment of the final aortogram and compared the results of the Myval THV series with the Sapien and Evolut THV series. Additionally, the analysis correlated the AR determined by echocardiography at discharge visit with the RF determined by videodensitometry immediately post-TAVI.

The main findings of this study were: 1) The QVDA assessment of the Myval THV series showed significantly smaller absolute RF and smaller rates of moderate/severe AR than in the Evolut THV series but had comparable results to the Sapien THV series. 2) After BPD in the Myval and Sapien THV series, there was no longer residual AR greater than 17 %, in contrast to Evolut THV series. 3) The QVDA achieved reliable ability in detecting none/trace AR and aortography was considered the most used imaging modality for the decision of BPD. 4) Pre-dilatation and presence of hypertension were identified as predictors for moderate/severe AR with QVDA.

The QVDA analysis has been validated in vitro [12,13] and in-vivo [23] and as well in the clinical setting and compared to transoesophageal echocardiography (TEE), transthoracic (TTE) and regurgitation derived from Magnetic Resonance [15,19]. Several previous studies have established a cut-off point of RF > 17 % as a threshold corresponding to moderate/severe AR on echocardiography [10,14,15,22], with a study identifying this threshold as a potential predictor of one-year mortality following TAVI [21]. Moreover, the usage of diastolic delta with videodensitometry led to an improvement in predicting PVL post-TAVI [24]. In this post-hoc analysis of the LANDMARK trial, 2.0 % of patients in the Myval THV series, 4.0 % in the Sapien THV series, and 8.0 % in the Evolut THV series had an RF > 17 % in the final aortogram. If left uncorrected, this may serve as an indicator of increased long-term mortality risk.

### 4.1 Yield of videodensitometry analysis in the LANDMARK

The analyzability rate of aortograms in this study (79.6 %), was higher than that reported in previous prospective and retrospective studies [14,15,17,24] but lower than other three studies [11,25,26]. This post-hoc analysis showed that the most common reasons for non-analyzability were breathing motion, coronary artery shadowing overlapping the region of interest (ROI), and poor image quality.

This analyzability rate surpasses that reported by Modolo et al. (2020), who assessed AR using quantitative videodensitometry in 3,976 participants and achieved an analyzability rate of 58.3 %, with overlapping of the ROI with the descending aorta and deep breathing identified as the primary causes of non-analyzability [15]. However, the analyzability of aortograms in the LANDMARK trial was lower than in the OVAL study [11] and the study by Tateishi et al. at Yamaguchi University Hospital [25], which reported analyzability rates of 92 % and 100 %, respectively. Tateishi et al. further divided participants into two groups, with one group receiving both a standard protocol and preprocedural MSCT for optimal angiographic projection planning, achieving 100 % analyzability. This underscores the importance of preprocedural MSCT in improving aortogram analyzability.

Although the 79.6 % analyzability rate is not optimal, the study's randomized controlled trial design ensures an equal distribution of participants across THV groups, allowing for an objective and balanced QVDA assessment in each arm.

### 4.2 Correlation between QVDA and echocardiography

The presence and severity of AR, as assessed by QVDA, has been correlated with echocardiographic findings at discharge and with cardiac magnetic resonance imaging within one-month post-procedure [18,21]. Our findings demonstrated a reasonable correlation between QVDA and echocardiography in identifying true-negative AR cases. Specifically, nearly two-thirds (67.7 %) of patients with none/trace AR on echocardiography had an RF < 6 %, while 42 % of those with moderate/severe AR on echocardiography had an RF > 17 %. However, QVDA showed limited sensitivity in detecting positive AR (sensitivity: 42.1 %, PPV: 45.6 %), which could hinder the detection of those cases with moderate/severe AR on echocardiography. However, QVDA usage gives clinical insight for identifying patients with none/trace AR if the RF < 6 %, and helps ensure that the patient underwent TAVI without having significant AR.

A retrospective observational study of patients who underwent TAVI reported that 50.2 % of those classified as having none/trace AR on echocardiography had an RF < 6 %, while 70 % of patients with moderate/severe AR had an RF > 17 % [27]. Findings from the LANDMARK trial further support QVDA’s reliability in identifying none/trace AR on echocardiography, showing moderate specificity (specificity: 70.8 %, NPV: 67.7 %), which helps with echocardiography in identifying true negative cases of AR. These results affirm that the QVDA can be used as a primary and pragmatic periprocedural imaging modality post-TAVI, either offline or online [11,18].

### 4.3 Difference in QVDA among the three valves

The QVDA of the three THV series showed that the Myval THV series had a median (Q1, Q3) RF of 3.0 (1.0, 7.0), comparable to the Sapien THV series (median (Q1, Q3) = 3.0 (1.0, 7.0), P_Myval vs Sapien_ = 0.8997), but significantly lower than the Evolut THV series (median (Q1, Q3) = 5.0 (1.0, 10.0), P_Myval vs Evolut_ = 0.001). Additionally, the incidence of moderate/severe AR in the Myval THV series (2 %) was numerically lower than in the Sapien THV series (4 %, P_Myval vs Evolut_ = 0.23) and significantly lower than in the Evolut THV series (8 %, P_Myval vs Evolut_ = 0.0057).

A study analyzing AR using quantitative videodensitometry across different THV types reported an RF > 17 % in 5.3 % of Evolut PRO THV cases and 8.8 % of Evolut R THV cases [15), aligning with the LANDMARK trial findings for the Evolut THV series. Conversely, the Sapien THV series demonstrated a higher incidence of RF > 17 %, with 8.3 % in SAPIEN 3 and 10.9 % in SAPIEN XT [15]. The lower percentage of moderate/severe AR observed in the Sapien THV series in the LANDMARK trial, compared to this previous study, may be attributed to advancements in the newer-generation Sapien THV, which have contributed to a reduction in AR following TAVI.

Furthermore, a study by Elkoumy et al. (2023) evaluated 122 final aortograms of the Myval Octacor THV, achieving an analyzability rate of 84.4 %. The study reported a RF of 2 %, with only two patients (1.9 %) exhibiting moderate/severe AR on aortogram [26]. This finding of moderate/severe AR aligns with the LANDMARK trial results (2 %) and with other study comparing the Myval THV series with two types of Sapien THV series, which reported a moderate/severe AR rate of 2.8 % in the Myval THV [22].

### 4.4 QVDA as guidance for the performance of BDP

Immediately post-TAVI implantation, the operator may perform balloon post-dilation (BPD) if aortic regurgitation (AR) is detected, aiming to optimize prosthesis expansion, improve the mean aortic gradient, and reduce AR. However, BPD carries potential risks, including an increased likelihood of stroke and annular rupture, and there are currently no formal guidelines for its use during TAVI [27].

The LANDMARK trial identified aortography as the primary imaging modality for performing BPD, which was performed in 118 patients. This accounted for 10 % (n = 38/384) in the Myval THV series and 21 % (n = 80/384) in the contemporary THV series (Sapien: 9.9 % (n = 19/192), Evolut: 31.8 % (n = 61/192)). Across all three groups, RF significantly decreased following BPD. Notably, no patients in the Myval THV series had RF > 17 % after BPD, whereas 5.9 % (n = 1) in the Sapien group and 8.5 % (n = 4) in the Evolut group still exceeded this threshold. This finding was initially reported by Miyazaki et al. (2018), who demonstrated that videodensitometry helped the decision-making process of BPD, leading to a significant reduction in RF after BPD [28]. Their study confirmed the effectiveness of BPD in reducing the severity of aortic regurgitation (AR) and lowering RF below the 17 % threshold, and thereby decreasing the risk impacting the long-term mortality (1-year mortality) which had been previously suggested in a retrospective analysis of a Brazilian registry [17].

### 4.5 Independent determinants of AR

In the LANDMARK trial, multivariate analysis revealed that pre-dilatation was significantly associated with moderate/severe AR on the final aortogram, whereas hypertension appeared to have a protective effect. However, univariate analysis showed aortic annulus area, LVOT area, total aortic calcium, predilatation and hypertension as independent predictors for moderate/severe AR. An earlier study reported leaflet calcification, larger THV size, and implantation depth as independent predictors for PVL [29].

### 4.6 Strengths and limitations

This post-hoc analysis of the LANDMARK trial provides comprehensive insights into QVDA of AR, achieving a reasonable analyzability rate of 79.6 %, which compares favorably to previous observational studies using a prospective protocol. Additionally, as a randomized controlled trial, the study ensures an equal distribution of participants across THV groups, allowing for an objective and balanced QVDA assessment in each arm. Moreover, this design also overcomes limitations seen in prior studies, which were often constrained by observational study design or low aortogram analyzability rates.

This study has limitations because it didn’t examine the relationship between videodensitometry findings and clinical outcomes—a correlation that could offer essential insights into mortality prediction. Therefore, long-term follow-up of this randomized trial is necessary to determine whether the modest yet statistically significant differences in post-procedural aortic regurgitation detected by videodensitometry will impact later clinical outcomes. Also, the monoplane and bidimensional aortography doesn’t allow a circumferential localization of the site of PVL or the differentiation between a central regurgitation and a PVL. Also, Sapein and Myval are intra-annular THVs, while Evolut is a supra-annular THV; that’s why the injection technique between THVs is different. Furthermore, although the randomization process ensures the balanced distribution of baseline and procedural characteristics, we cannot entirely exclude the potential for other confounding factors to have influenced the results.

## 5 Conclusion

In the Myval THV series, the QVDA analysis immediately post-implantation showed a comparable RF to the Sapien THV series, but significantly higher than the Evolut THV series. Furthermore, a reduction of RF in all arms of THV following BPD has been documented. The QVDA assessment of AR showed good ability to detect none/trace AR on echocardiography and can be used in conjunction with echocardiography assessment during the procedure to help in the decision-making process of performing a palliative or curative correction of the post-TAVI AR that can be detrimental in the long-term outcomes of a TAVI procedure.

Trial Registration number

NCT04275726 (ClinicalTrials.gov).

## CRediT authorship contribution statement

**Elfatih A. Hasabo:** Writing – review & editing, Writing – original draft, Software, Methodology, Formal analysis, Data curation. **Niels van Royen:** Writing – review & editing, Investigation. **Ignacio J Amat-Santos:** Writing – review & editing, Investigation. **Martin Hudec:** Writing – review & editing, Investigation. **Matjaz Bunc:** Writing – review & editing, Investigation. **Alexander IJsselmuiden:** Writing – review & editing, Investigation. **Peep Laanmets:** Writing – review & editing, Investigation. **Daniel Unic:** Writing – review & editing, Investigation. **Bela Merkely:** Writing – review & editing, Investigation. **Renicus S Hermanides:** Writing – review & editing, Investigation. **Vlasis Ninios:** Writing – review & editing, Investigation. **Marcin Protasiewicz:** Writing – review & editing, Investigation. **Benno J W M Rensing:** Writing – review & editing, Investigation. **Pedro L Martin:** Writing – review & editing, Investigation. **Fausto Feres:** Writing – review & editing, Investigation. **Manuel De Sousa Almeida:** Writing – review & editing, Investigation. **Eric van Belle:** Writing – review & editing, Investigation. **Axel Linke:** Writing – review & editing, Investigation. **Alfonso Ielasi:** Writing – review & editing, Investigation. **Matteo Montorfano:** Writing – review & editing, Investigation. **Mark Webster:** Writing – review & editing, Investigation. **Konstantinos Toutouzas:** Writing – review & editing, Investigation. **Emmanuel Teiger:** Writing – review & editing, Investigation. **Francesco Bedogni:** Writing – review & editing, Investigation. **Michiel Voskuil:** Writing – review & editing, Investigation. **Manuel Pan:** Writing – review & editing, Investigation. **Oskar Angerås:** Writing – review & editing, Investigation. **Won-Keun Kim:** Writing – review & editing, Investigation. **Jürgen Rothe:** Writing – review & editing, Investigation. **Ivica Kristić:** Writing – review & editing, Investigation. **Vicente Peral:** Writing – review & editing, Investigation. **Ben J.L. Van den Branden:** Writing – review & editing, Investigation. **Ashokkumar Thakkar:** Writing – review & editing, Methodology, Conceptualization. **Udita Chandra:** Writing – review & editing, Investigation. **Cagri Ayhan:** Writing – review & editing, Investigation. **Dina Neiroukh:** Writing – review & editing, Software, Resources. **Mahmoud Y. Nosir:** Writing – review & editing, Software, Methodology. **Magdi S. Yacoub:** Writing – review & editing, Investigation. **Sanaa Ali:** Writing – review & editing, Software, Methodology. **Mohamad Altamimi:** Writing – review & editing, Software, Resources. **Hesham Elzomor:** Writing – review & editing, Software, Resources, Methodology. **Patrick W Serruys:** Writing – review & editing, Project administration, Methodology. **Andreas Baumbach:** Writing – review & editing, Project administration, Methodology, Investigation. **Osama Soliman:** Writing – review & editing, Supervision, Software, Project administration, Methodology, Investigation, Conceptualization.

## Funding

This trial is sponsored by Meril Life Sciences Pvt. Ltd., India.

## Declaration of competing interest

The authors declare the following financial interests/personal relationships which may be considered as potential competing interests: [Osama Soliman reports research grants from Biosensors, Boston Scientific, Cardiawave and Meril Life Sciences. P.W. Serruys reports consultancy fees from SMT, Novartis, Meril Life Sciences, and Philips. N. van Royen reports grant funding and personal fees from Abbott; grants from Philips, Biotronik, and Medtronic; and speaker fees from MicroPort, Bayer, and RainMed Medical outside the submitted work. I.J. Amat-Santos reports being a proctor for Medtronic, Boston Scientific, and Meril Life Sciences. A. Ijsselmuiden reports institutional fees from Medtronic and Abbott; consulting fees from Meril Life Sciences, Angiocare, Abbott, Philips, and Translumina. P. Laanmets received travel support from Meril Life Sciences to attend the conference. D. Unic reports payment/honoraria from Meril Life Sciences, Medtronic and abbott; and a member of the Medtronic EMEA surgical advisory board. B. Merkely reports institutional grants and speaker fees from Boehringer Ingelheim, DUKE Clinical Institute, and Novartis; institutional fees from Biotronik and Eli Lilly; direct personal payment from Daiichi Sankyo; national leader for Librexia program, New Amsterdam trial, DAPA ACT HF-TIMI 68 trial, FINEARTS-HF trial, REALIZE-K trial, SOS-AMI trial, DELIVER trial, GARDEN-TIMI 74 trial, ENDEAVOR trial, EMPACT-MI trial, CARDINAL-HF trial; rector of Semmelweis University, Director and chair of the Heart and Vascular Center of Semmelweis University. R.S. Hermanides reports speaker fees from Novartis, Edwards Life Sciences, Meril and Abbott vascular outside the submitted work. P. Martin reports proctorship grant from Meril Life Sciences; payment or honoraria for lectures, presentations from Meril Life Sciences, Boston Scientific Iberica, Abbott; Advisory board member for Medtronic Spain. M. De Sousa Almeida reports lecture fees from Medtronic and Novartis; travel support from Medtronic, Terumo and Boston Scientific. A. Linke received grants from Edward Lifesciences and Novartis; speaker honoraria from Edward Lifesciences, Boston Scientific, AbioMed, Pfizer, Astra Zeneca, Boehringer, Abbott, MSD, Corvia, Daiichi, and Meril; travel support from Meril, AbioMed and abbott; Stock option holder with Picardia, Transverse Medical and Filterlex. Alfonso Ielasi reports consulting fees, payment/honoraria for lectures, presentations from Meril Life Sciences, Sahajanand Medical Technologies and Cardionovum. K. Toutouzas reports proctorship with Abbott, Meril and Medtronic; consulting fee from Gore Medical; Board member Hellenic Society of Cardiology. F. Bedogni reports proctorship and consulting fees from Meril Life Sciences and Medtronic. Dolores Mesa Rubio reports minor lecture fees from Edwards and Abbott. O. Angerås reports proctorship and speaker fees from Meril Life and Abbott Medical; speaker fees from Medtronic; research grant from Abbott. W.-K. Kim reports honoraria or consultancy fees from Edwards Lifesciences, Boston Scientific, Meril Life Sciences, JenaValve, Abbott and P&F; advisory board member for P&F. J. Rothe reports personal fees for consulting/proctoring from Meril Life Sciences, Medtronic, Abbott and Qatna; and travel support for attending meetings from Meril Life Sciences, Edwards Lifesciences, Abbott, Medtronic, and Boston Scientific. A Thakkar, and U Chandra are full employees of Meril Life Sciences. P.W. Serruys reports consultancy fees from SMT, Novartis, Meril Life Sciences, and Philips. A. Baumbach reports consultation fees from Meril Life Sciences, Biotronik and Jenavalve; Lecture fees or honoraria from Biotronik; participation in DSMB for Pi Cardia and Faraday. The other authors have no conflicts of interest to declare.].

## Data Availability

The data associated with this publication will be made available upon reasonable request to the corresponding author.
