# Supplementary material for: Quantitative videodensitometric assessment of aortic regurgitation in Myval, Sapien, and Evolut THV series: Results from the LANDMARK trial
Source: Int J Cardiol Heart Vasc. 2025 Sep 29;61:101804. doi: 10.1016/j.ijcha.2025.101804 (PMC12512974; doi:10.1016/j.ijcha.2025.101804)
Supplement: Supplementary Data 1 [file mmc1.docx]

# **Supplementary Figure 1**: Aortic regurgitation grading, in the Myval, Sapien and Evolut THV series based on the final aortogram post-TAVI analyzed by QVDA and categorized in none /trace, mild, and moderate/severe AR.

| **RF cutoff-point** | **Myval THV series (N=295)** | **Sapien THV series (N=151)** | **Evolut THV series (N=150)** | **P-values (Overall)** | **P-value (Myval vs Sapien)** | **P-value (Myval vs Evolut)** |
| --- | --- | --- | --- | --- | --- | --- |
| **<6%** | 203 (68.8) | 100 (66.2) | 77 (51.3) | **0.0012** | 0.6548 | **0.0001** |
| **6—17%** | 86 (29.2) | 45 (29.8) | 61 (40.7) | **0.0373** | 0.9741 | **0.0196** |
| **> 17%** | 6 (2.0) | 6 (4.0) | 12 (8.0) | **0.0103** | 0.2333 | **0.0057** |

# **Supplementary Table 1:** Reasons for performing balloon post-dilatation and the imaging modalities used for the decision.

| **Reason for post-dilatation** | **All=118** | **Myval THV series (N=38)** | **Sapien THV series (n=19)** | **Evolut THV series (n=61)** | **P-value**  **(Myval Vs Sapien)** | **P-value (Myval Vs Evolut)** |
| --- | --- | --- | --- | --- | --- | --- |
| Paravalvular leak | 75 (63.56) | 27 (71.05) | 12 (63.16) | 36 (59.02) | 0.76 | 0.32 |
| Suboptimal valve expansion | 26 (22.03) | 7 (18.42) | 2 (10.53) | 17 (27.87) | 0.70 | 0.41 |
| Paravalvular leak and suboptimal valve expansion | 14 (11.86) | 4 (10.53) | 4 (21.05) | 6 (9.84) | 0.42 | 1.00 |
| Residual pressure gradient | 2 (1.69) | 0 (0.00) | 1 (5.26) | 1 (1.64) | 0.33 | 1.00 |
| Suboptimal valve expansion and residual pressure gradient | 1 (0.85) | 0 (0.00) | 0 (0.00) | 1 (1.64) | - | 1.00 |
| **Modality for decision of performing post-dilatation** | | | | | | |
| Aortography/Cine | 90 (76.27) | 35 (92.11) | 11 (57.89) | 44 (72.13) | 0.004 | 0.03 |
| Aortography/Cine and Transthoracic echocardiography | 10 (8.47) | 2 (5.26) | 4 (21.05) | 4 (6.56) | 0.09 | 1.00 |
| Transesophageal echocardiography | 5 (4.24) | 1 (2.63) | 0 (0) | 4 (6.56) | 1.00 | 0.65 |
| Transthoracic echocardiography | 4 (3.39) | 0 (0.00) | 3 (15.79) | 1 (1.64) | 0.03 | 1.00 |
| Hemodynamics | 3 (2.54) | 0 (0.00) | 0 (0) | 3 (4.92) | - | 0.28 |
| Aortography/Cine, Transthoracic echocardiography and Hemodynamics | 2 (1.69) | 0 (0.00) | 0 (0) | 2 (3.28) | - | 0.52 |
| Aortography/Cine and Transesophageal echocardiography | 2 (1.69) | 0 (0.00) | 1 (5.26) | 1 (1.64) | 0.3333 | 1.00 |
| Aortography/Cine and Hemodynamics | 1 (0.85) | 0 (0.00) | 0 (0) | 1 (1.64) | - | 1.00 |
| Transthoracic echocardiography and Hemodynamics | 1 (0.85) | 0 (0.00) | 1 (5.26) | 0 (0) | 0.33 | 1.00 |

# **Supplementary Table 2:** Reasons for non-analysability of QVDA (n=153).

| **Reason for non-analyzability (Multiple answer question)** | **Overall (n=153)** |
| --- | --- |
| Breathing motion | 36 (24%) |
| Coronary arteries shadowing overlapping ROI | 26 (17%) |
| Poor image quality | 26 (17%) |
| Descending aorta overlapping LVOT | 24 (16%) |
| Significant cardiac motion | 18 (12%) |
| Descending aorta overlapping ascending aorta (reference area) | 16 (10%) |
| Cine rate <15 | 15 (9.8%) |
| TEE probe leads, electrodes or other objects overlapping ROI. | 13 (8.5%) |
| Moving diaphragm overlapping ROI. | 11 (7.2%) |
| Lung field affecting videodensitometry | 10 (6.5%) |
| Insufficient image acquisition (less than 2 cardiac cycles before contrast injection). | 7 (4.6%) |
| Final deployment of valve not filmed | 7 (4.6%) |
| Significant Mitral calcification affecting Video densitometry | 7 (4.6%) |
| Inadequate contrast filling | 4 (2.6%) |
| Catheter position too deep | 4 (2.6%) |
| Guidewire in left ventricle | 2 (1.3%) |
| Catheter in left ventricle. | 2 (1.3%) |
| Less than 30 frames | 1 (0.7%) |
| Table and/or patient motion. | 1 (0.7%) |
| Insufficient Image acquisition (less than 3 cardiac cycles after contrast Injection). | 1 (0.7%) |
| Digital clock angio recording not activated | 1 (0.7%) |
| Others (Regurgitation results cannot be calculated via the software, only images received, ECG not recognized) | 8 (5.2%) |

# **Supplementary Table 3:** Site-wise frequency of non-analyzability of QVDA (n=153).

| **Site number** | **No. of non-analyzable QVDA (n/N)** | **Site number** | **No. of non-analyzable QVDA (n/N)** |
| --- | --- | --- | --- |
| Site #1 | 17/96 (17.7%) | Site #17 | 2/6 (33.3%) |
| Site #2 | 1/23 (4.3%) | Site #18 | 2/7 (28.6%) |
| Site #3 | 8/52 (15.4%) | Site #19 | 6/7 (85.7%) |
| Site #4 | 11/110 (10.0%) | Site #20 | 6/23 (26.1%) |
| Site #5 | 4/29 (13.8%) | Site #21 | 5/12 (41.7%) |
| Site #6 | 4/22 (18.2%) | Site #22 | 2/5 (40.0%) |
| Site #7 | 13/71 (18.3%) | Site #23 | 2/5 (40.0%) |
| Site #8 | 0/1 (0%) | Site #24 | 0/3 (0%) |
| Site #9 | 3/21 (14.3%) | Site #25 | 8/43 (18.6%) |
| Site #10 | 2/9 (22.2%) | Site #26 | 1/2 (50.0%) |
| Site #11 | 20/80 (25.0%) | Site #27 | 1/2 (50.0%) |
| Site #12 | 2/7 (25.6%) | Site #28 | 4/10 (40.0%) |
| Site #13 | 1/8 (12.5%) | Site #29 | 4/11 (36.4%) |
| Site #14 | 7/39 (18.0%) | Site #30 | 4/5 (80.0%) |
| Site #15 | 4/15 (26.7%) | Site #31 | 4/22 (18.2%) |
| Site #16 | 5/22 (22.7%) |  |  |

Data was presented as number (Percentage)

# **Supplementary Table 4:** Hemodynamic, procedural and lesional characteristics of the patients with analyzable final aortograms, randomized in the three THV series.

| **Baseline Characteristics** | **Myval THV series (N=295)** | Sapien THV series (N=151) | Evolut THV series (N=150) |
| --- | --- | --- | --- |
|  |  |  |  |
| **Echocardiography findings** |  |  |  |
| Aortic valve mean gradient, mm Hg | 39.7 ± 14.3 (n=287) | 38.6 ± 14.9 (n=145) | 38..0 ± 12.8 (n=144) |
| Aortic valve peak gradient, mm Hg | 64.9 ± 22.1 (n=287) | 62.2 ± 22.2 (n=145) | 62.8 ± 20.1 (n=144) |
| Aortic valve peak velocity, m/s | 4.0 ± 0.7 (n=287) | 3.9 ± 0.7 (n=145) | 3.9 ± 0.6 (n=144) |
| Aortic valve area, cm^2^ | 0.74 ± 0.23 (n=285) | 0.69 ± 0.2 (n=142) | 0.74 ± 0.23 (n=142) |
| Left ventricular ejection fraction, % | 58.8 ± 10.4 (n=207) | 57.0 ± 10.6 (n=112) | 57.4 ± 9.4 (n=113) |
| Systolic pulmonary artery pressure, mm Hg | 33.1 ± 14.1 (n=224) | 32.9 ± 12.6 (n=122) | 35.6 ± 12.9 (n=110) |
| Aortic valve EOA, cm^2^ | 0.74 ± 0.23 (n=285) | 0.69 ± 0.2 (n=142) | 0.74 ± 0.23 (n=142) |
| **Aortic regurgitation** | **n=279** | **n=146** | **n=142** |
| None/Trace | 103 (36.9) | 52 (35.6) | 44 (31.0) |
| Mild | 143 (51.3) | 79 (54.1) | 78 (54.9) |
| Moderate | 29 (10.4) | 15 (10.3) | 20 (14.1) |
| Severe | 4 (1.4) | 0 (0.0) | 0 (0.0) |
| Moderate or higher aortic regurgitation | 33 (11.8) | 15 (10.3) | 20 (14.1) |
| **Mitral regurgitation** | **n=281** | **n=147** | **n=140** |
| None/Trace | 102 (36.3) | 46 (31.3) | 43 (30.7) |
| Mild | 145 (51.6) | 75 (51.0) | 84 (60.0) |
| Moderate | 26 (9.3) | 20 (13.6) | 12 (8.6) |
| Severe | 8 (2.9) | 6 (4.1) | 1 (0.7) |
| Moderate or higher mitral regurgitation | 34 (12.1) | 26 (17.7) | 13 (9.3) |
| **Tricuspid regurgitation** | **n=268** | **n=143** | **n=133** |
| None/Trace | 134 (50.0) | 58 (40.6) | 58 (43.6) |
| Mild | 109 (40.7) | 63 (44.1) | 58 (43.6) |
| Moderate | 17 (6.3) | 15 (10.5) | 12 (9.0) |
| Severe | 8 (3.0) | 7 (4.9) | 5 (3.8) |
| Moderate or higher tricuspid regurgitation | 25 (9.3) | 22 (15.4) | 17 (12.8) |
| **Bicuspid valve** | **n=18 (%)** | **n=14(%)** | **n=7 (%)** |
| Type 0 | 4 (22.2) | 0 (0.0) | 0 (0.0) |
| Type 1a | 11 (61.1) | 11 (78.6) | 6 (85.7) |
| Type 1b | 3 (16.7) | 3 (21.4) | 1 (14.3) |
| **Procedure** |  |  |  |
| **Vascular access** | **n=295** | **n=251** | **n=150** |
| Transfemoral approach, (%) | 295 (100.0) | 150(99.3) | 150 (100.0) |
| Subclavian approach, (%) | - | 1(0.7) | - |
| Transaortic approach, (%) | - | - | - |
| Predilatation | 124 (42.0) | 48 (31.8) | 64 (42.7) |
| **CT findings** |  |  |  |
| Aortic annulus perimeter, mm | 77.7 ± 6.5 (n=295) | 77.9 ± 6.8 (n=151) | 78.0 ± 6.0 (n=150) |
| Aortic annulus area, mm^2^ | 468.6 ± 77.8 (n=295) | 471.9 ± 83.1 (n=151) | 471.9 ± 73.5 (n=150) |
| Small annulus (aortic annulus area ≤430 mm^2^) (%) | 97 (32.88) (n=295) | 49 (32.45) (n=151) | 43 (28.67) (n=150) |
| Total calcium aortic valve, mm^3^univariate ,anova | 1029.5 ± 729.8 (n=295) | 968.8 ± 675.8 (n=151) | 978.4 ± 623.3 (n=150) |
| **Aortic annulus characteristics** |  |  |  |
| Min diameters, mm | 21.7 ± 2.1 (n=295) | 21.8 ± 2.3 (n=151) | 21.9 ± 2.1 (n=150) |
| Max diameter, mm | 27.4 ± 2.4 (n=295) | 27.5 ± 2.5 (n=151) | 27.5 ± 2.3 (n=150) |
| Mean diameter, mm | 24.6 ± 2.1 (n=295) | 24.6 ± 2.2 (n=151) | 24.7 ± 2.0 (n=150) |
| Perimeter derived diameter, mm | 24.7 ± 2.1 (n=295) | 24.8 ± 2.2 (n=151) | 24.8 ± 1.9 (n=150) |
| Annulus area derived diameter, mm^2^ | 24.4 ± 2.0 (n=295) | 24.4 ± 2.2 (n=151) | 24.4 ± 1.9 (n=150) |
| Perimeter, mm | 77.7 ± 6.5 (n=295) | 77.9 ± 6.8 (n=151) | 78.0 ± 6.0 (n=150) |
| Area, mm^2^ | 468.6 ± 77.8 (n=295) | 471.9 ± 83.1 (n=151) | 471.9 ± 73.5 (n=150) |
| **Calcification (Quantitative)** |  |  |  |
| AV calcification volume, mm^3^ (Quantitative) | 1029.5 ± 729.8 (n=295) | 968.8 ± 675.8 (n=151) | 978.4 ± 623.3 (n=150) |
| **Calcification (Qualitative)** |  |  |  |
| **AV calcification severity (qualitative)** | **n=294** | **n=151** | **n=150** |
| No AV calcification | 0 (0.0) | 3 (2.0) | 2 (1.3) |
| Mild AV calcification | 45 (15.3) | 29 (19.2) | 25 (16.7) |
| Moderate AV calcification | 116 (39.5) | 56 (37.1) | 53 (35.3) |
| Severe AV calcification | 133 (45.2) | 63 (41.7) | 70 (46.7) |
| **LVOT calcification severity (qualitative)** | **n=280** | **n=147** | **n=144** |
| No LVOT calcification | 276 (98.6) | 146 (99.3) | 143 (99.3) |
| Mild LVOT calcification | 3 (1.1) | 1 (0.7) | 1 (0.7) |
| Moderate LVOT calcification | 1 (0.4) | 0 (0.0) | 0 (0.0) |
| Severe LVOT calcification | 0 (0.0) | 0 (0.0) | 0 (0.0) |

Data was presented as number (Percentage), Mean ± SD, Median (Q1-Q3)

# **Supplementary Table 5:** Baseline demographic and lesional characteristics of the study population with analyzable videodensitometry vs non-analyzable videodensitometry.

| **Baseline Characteristics** | **Analyzable videodensitometry**  **(N=596)** | **Non-analyzable videodensitometry**  **(N=153)** | **P-value** | **95% CI** |
| --- | --- | --- | --- | --- |
| Age, (year) | 80.2 ± 5.6 (n=596) | 80.0 ± 5.8 (n=153) | 0.7061 | 0.2 ( -0.82, 1.22) |
| Female, (%) | 289 (48.5) | 74 (48.9) | 1.0000 | -0.4 (-8.88, 9.12) |
| Body mass index (kg/m^2^) | 27.9 ± 4.7 (n=596) | 29.0 ± 5.3 (n=153) | **0.0248** | **-1.1 ( -2.02, -0.18)** |
| Body surface area (m^2^) | 1.9 ± 0.2 (n=596) | 1.9 ± 0.2 (n=153) | 0.2068 | 0.0 ( -0.04, 0.04) |
| **Society of Thoracic Surgeons score** | 3.1 ± 2.3 (n=596) | 3.8 ± 2.6 (n=153) | 0.0069 | -0.7 ( -1.15, -0.25) |
| Low score (<4) | 462 (77.5) | 103 (67.3) | 1.0000 |  |
| Intermediate score (4–8) | 115 (19.3) | 37 (24.2) |  |  |
| High score (>8) | 19 (3.2) | 13 (8.5) |  |  |
| EuroSCORE II | 3.9 ± 8.6 (n=90) | 2.7 ± 1.7 (n=25) | 0.2044 | 1.2 (-0.7, 3.1) |
| **New York Heart Association (NYHA)** | **n=596** | **n=153** |  |  |
| Class I | 23 (3.9) | 5 (3.3) | 0.9323 |  |
| Class II | 263 (44.1) | 65 (42.5) |  |  |
| Class III | 284 (47.7) | 75 (49.0) |  |  |
| Class IV | 26 (4.4) | 8 (5.2) |  |  |
| **Medical history** |  |  |  |  |
| Hypercholesterolaemia | 64 (10.7) | 14 (9.2) | 0.6706 | 1.5 (-4.02, 7.20) |
| Hypertension | 388 (65.1) | 110 (71.9) | 0.1356 | -6.8 (-15.29, 1.70) |
| Current smoker | 147 (24.7) | 37 (24.2) | 0.9855 | 0.5 (-7.55, 8.51) |
| Alcohol consumption | 136 (22.8) | 25 (16.3) | 0.1031 | 6.5 (-0.69, 13.65) |
| Current Diabetes Mellitus, (%) | 160 (26.9) | 57 (37.3) | 0.0150 | -10.4 (-19.27, -1.55) |
| Stroke | 13 (2.2) | 6 (3.9) | 0.2467 | -1.7 (-5.44, 1.96) |
| Atrial fibrillation, (%) | 147 (24.7) | 42 (27.5) | 0.5461 | -2.8 (-11.07, 5.50) |
| Chronic obstructive pulmonary disease, (%) | 58 (9.7) | 23 (15.0) | 0.0823 | -5.3 (-11.85, 1.25) |
| Myocardial infarction, (%) | 40 (6.7) | 8 (5.2) | 0.6291 | 1.5 (-2.99, 5.95) |
| Coronary artery disease, (%) | 90 (15.1) | 22 (14.4) | 0.9234 | 0.7 (-5.95, 7.39) |
| Prior coronary artery bypass grafting, (%) | 28 (4.7) | 5 (3.3) | 0.5837 | 1.4 (-2.27, 5.13) |
| Prior percutaneous coronary intervention, (%) | 38 (6.4) | 15 (9.8) | 0.1942 | -3.4 (-8.94, 2.09) |
| Prior balloon aortic Valvuloplasty, % | 4 (0.7) | 0 (0.0) | 1.0000 | 0.7 (-0.40, 1.74) |
| Cerebrovascular accident, (%) | 4 (0.7) | 2 (1.31) | 0.3205 | -0.6 (-2.96, 1.69) |
| Porcelain aorta or hostile chest Procedural Characteristics) (%) | 0 (0.00) | 0 (0.0) | - | - |
| Peripheral vascular disease, (%) | 5 (0.8) | 1 (0.7) | 1.0000 | 0.1 (-1.47, 1.84) |
| Overall frailty, (%) | 85 (14.3) | 19 (12.4) | 0.9393 | 1.9 (-4.50, 8.19) |
| Pulmonary hypertension, (%) | 12 (2.0) | 3 (2.0) | 1.0000 | 0.0 (-2.47, 2.57) |
| Permanent pacemaker, (%) | 22 (3.7) | 7 (4.6) | 0.7867 | -0.9 (-4.94, 3.17) |
| Left bundle branch block, (%) | 25 (4.2) | 3 (2.0) | 0.2889 | 2.2 (-0.90, 5.37) |
| Right bundle branch block, (%) | 28 (4.7) | 11 (7.2) | 0.3014 | -2.5 (-7.33, 2.35) |
| Estimated glomerular filtration rate <60 mL/min | 266 (46.8) (n=568) | 72 (51.43) (n=140) | 0.3783 | -4.6 (-14.28, 5.09) |
| Estimated glomerular filtration rate <30 mL/min | 79 (13.9) (n=568) | 24 (17.14) (n=140) | 0.4018 | -3.2 (-10.54, 4.07) |
| **Echocardiography findings** |  |  |  |  |
| Aortic valve mean gradient, mm Hg | 39.0 ± 14.1 (n=576) | 40.7 ± 12.6 (n=148) | 0.1589 | -1.7 (-4.03, 0.63) |
| Aortic valve peak gradient, mm Hg | 63.7 ± 21.6 (n=576) | 66.3 ± 19.3 (n=148) | 0.1530 | -2.6 (-6.17, 0.97) |
| Aortic valve peak velocity, m/s | 3.9 ± 0.7 (n=576) | 4.0 ± 0.6 (n=148) | 0.0927 | -0.1 (-0.21, 0.01) |
| Aortic valve area, cm^2^ | 0.73 ± 0.22 (n=569) | 0.72 ± 0.21 (n=144) | 0.8280 | 0.01 (-0.03, 0.05) |
| Left ventricular ejection fraction, % | 58.0 ± 10.2 (n=432) | 57.9 ± 9.6 (n=98) | 0.9415 | 0.1 (-2.03, 2.23) |
| Systolic pulmonary artery pressure, mm Hg | 33.7 ± 13.4 (n=456) | 35.6 ± 15.9 (n=118) | 0.2244 | -1.9 (-5.02, 1.22) |
| Aortic valve EOA, cm^2^ | 0.73 ± 0.22 (n=569) | 0.72 ± 0.21 (n=144) | 0.8280 | 0.01 (-0.03, 0.05) |
| **Aortic regurgitation** | **n=567** | **n=149** |  |  |
| None/Trace | 199 (35.1) | 51 (34.2) | 0.9780 |  |
| Mild | 300 (52.9) | 79 (53.0) |  |  |
| Moderate | 64 (11.3) | 18 (12.1) |  |  |
| Severe | 4 (0.7) | 1 (0.7) |  |  |
| Moderate or higher aortic regurgitation | 68 (12.0) | 19 (12.8) |  |  |
| **Mitral regurgitation** | **n=568** | **n=144** |  |  |
| None/Trace | 191 (33.6) | 56 (38.89) | 0.2296 |  |
| Mild | 304 (53.5) | 64 (44.44) |  |  |
| Moderate | 58 (10.2) | 19 (13.19) |  |  |
| Severe | 15 (2.6) | 5 (3.47) |  |  |
| Moderate or higher mitral regurgitation | 73 (12.9) | 24 (16.67) |  |  |
| **Tricuspid regurgitation** | **n=544** | **n=141** |  |  |
| None/Trace | 250 (46.0) | 60 (42.6) | 0.9036 | - |
| Mild | 230 (42.3) | 64 (45.4) |  |  |
| Moderate | 44 (8.1) | 12 (8.5) |  |  |
| Severe | 20 (3.7) | 5 (3.6) |  |  |
| Moderate or higher tricuspid regurgitation | 64 (11.8) | 17 (12.1) |  |  |
| **Bicuspid valve** | **n=39** | **n=13** |  |  |
| Type 0 | 4 (10.3) | 1 (7.7) | 0.8766 |  |
| Type 1a | 28 (71.8) | 9 (69.2) |  | - |
| Type 1b | 7 (18.0) | 3 (23.1) |  |  |
| **Procedure** |  |  |  |  |
| **Vascular access** |  |  |  |  |
| Transfemoral approach, (%) | 595 (99.8) (n=596) | 152 (99.3) (n=153) | 0.3670 | 0.5 (-1.24, 2.21) |
| Subclavian approach, (%) | 1 (0.2) (n=596) | 1 (0.7) (n=153) | 0.3670 | -0.5 (-2.21, 1.24) |
| Transaortic approach, (%) | 0 (0.0) | 0 (0.0) | -- | -- |
| Predilatation | 236 (39.6) | 68 (44.4) | 0.3188 | -4.8 (-14.06, 4.36) |
| **RF Median (Q1-Q3)** | 3 (1 - 8) (n=596) | - | - |  |
| <6% | 380 (63.8) | -- | -- |  |
| 6—17% | 192 (32.2) | -- | -- |  |
| > 17% | 24 (4.0) | -- | -- |  |
| **CT findings** |  |  |  |  |
| Aortic annulus perimeter, mm | 77.8 ± 6.4 (n=596) | 78.0 ± 7.0 (n=153) | 0.8436 | -0.2 (-1.42, 1.02) |
| Aortic annulus area, mm^2^ | 470.3 ± 78.0 (n=596) | 473.2 ± 82.5 (n=153) | 0.6957 | -2.9 (-17.39, 11.59) |
| Small annulus (aortic annulus area ≤430 mm2) | 189 (31.7) (n=596) | 50 (32.6) (n=153) | 0.8950 | -0.9 (-9.70, 7.76) |
| Total calcium aortic valve, mm^3^ | 1001.3 ± 690.2 (n=596) | 1035.2 ± 699.0 (n=153) | 0.5920 | -33.9 (-157.75, 89.95) |
| **Aortic annulus characteristics** | **n=596** | **n=153** |  |  |
| Min diameters, mm | 21.8 ± 2.1 (n=596) | 21.9 ± 2.0 (n=153) | 0.4016 | -0.1 (-0.46, 0.26) |
| Max diameter, mm | 27.4 ± 2.4 (n=596) | 27.5 ± 2.8 (n=153) | 0.9367 | -0.1 (-0.58, 0.38) |
| Mean diameter, mm | 24.6 ± 2.1 (n=596) | 24.7 ± 2.2 (n=153) | 0.6635 | -0.1 (-0.49, 0.29) |
| Perimeter derived diameter, mm | 24.8 ± 2.1 (n=596) | 24.8 ± 2.2 (n=153) | 0.8313 | 0.0 (-0.39, 0.39) |
| Annulus derived diameter, mm | 24.4 ± 2.0 (n=596) | 24.5 ± 2.2 (n=153) | 0.7685 | -0.1 (-0.48, 0.28) |
| Perimeter, mm | 77.8 ± 6.4 (n=596) | 78.0 ± 7.0 (n=153) | 0.8436 | -0.2 (-1.42, 1.02) |
| Area, mm^2^ | 470.3 ± 78.0 (n=596) | 473.2 ± 82.5 (n=153) | 0.6957 | -2.9 (-17.39, 11.59) |
| **Calcification (Qualitative)** |  |  |  |  |
| AV calcium volume (quantitative) | 1001.3 ± 690.2 (n=596) | 1035.2 ± 699.0 (n=153) | 0.5920 | -33.9 (-157.75, 89.95) |
| **AV calcification severity (qualitative)** | **n=595** | **n=153** |  |  |
| No AV calcification | 5 (0.8) | 1 (0.7) | 0.7732 |  |
| Mild AV calcification | 99 (16.6) | 28 (18.3) |  |  |
| Moderate AV calcification | 225 (37.8) | 51 (33.3) |  |  |
| severe AV calcification | 266 (44.7) | 73 (47.7) |  |  |
| **LVOT calcification severity (qualitative)** | **n=571** | **n=139** |  |  |
| No LVOT calcification | 565 (99.0) | 137 (98.6) | 0.7010 |  |
| Mild LVOT calcification | 5 (0.9) | 2 (1.4) |  |  |
| Moderate LVOT calcification | 1 (0.2) | 0 (0.0) |  |  |
| Severe LVOT calcification | 0 (0.0) | 0 (0.0) |  |  |
| Membranous septum length | 4.36 ± 2.38 (n=304) | 4.34 ± 2 (n=62) | **0.0594** | **0.02 ( -0.55, 0.59)** |

Data was presented as number (Percentage), Mean ± SD, Median (Q1-Q3)

# **Supplementary Table 6:** Baseline characteristics and clinical data of the study population with balloon post-dilatation (Myval vs Sapien vs Evolut)

| **Baseline Characteristics** | **Myval THV series (N=38)** | **Sapien THV series**  **(N=19)** | **Evolut THV series**  **(N=61)** | **P-value** | **95% CI** | |
| --- | --- | --- | --- | --- | --- | --- |
|  |  |  |  |  | **Myval vs Sapien** | **Myval vs Evolut** |
| Age, (year) | 80.8 ± 5.4 (n=38) | 80.7 ± 7.1 (n=19) | 79.6 ± 4.1 (n=61) | 0.4565 | 0.1 (-3.8, 4.0) | 1.2 (-0.9, 3.3) |
| Female, (%) | 20 (52.6) | 7 (36.8) | 35 (57.4) | 0.2937 | 15.8 (-15.0, 46.6) | -4.8 (-27.03, 17.5) |
| Body mass index (kg/m^2^) | 27.6 ± 3.8 (n=38) | 27.8 ± 2.5 (n=19) | 27.2 ± 4.2 (n=61) | 0.7919 | -0.2 (-2.0, 1.6) | 0.4 (-1.3, 2.1) |
| Body surface area (m^2^) | 3.4 ± 0.7 (n=38) | 3.6 ± 0.7 (n=19) | 3.5 ± 0.9 (n=61) | 0.6202 | -0.2 (-0.6, 0.2) | -0.1 (-0.4, 0.2) |
| **Society of Thoracic Surgeons score** | 3.7 ± 2.7 (n=38) | 3.3 ± 2.8 (n=19) | 3.3 ± 2.3 (n=61) | 0.7095 | 0.4 (-1.2, 2) | 0.4 (-0.7, 1.5) |
| Low score (<4) | 25 (65.8) | 16 (84.2) | 45 (73.8) | 0.3065 | - | - |
| Intermediate score (4–8) | 10 (26.3) | 1 (5.3) | 13 (21.3) |  |  |  |
| High score (>8) | 3 (7.9) | 2 (10.5) | 3 (4.9) |  |  |  |
| EuroSCORE II | 3.5 ± 1.8 (n=9) | 5.5 ± 5.6 (n=5) | 1.7 ± 0.6 (n=3) | 0.3096 | -2.0 (-9.1, 5.1) | 1.8 (-1.2, 4.8) |
| **New York Heart Association (NYHA)** | n=38 | n=19 | n=61 |  |  |  |
| Class I | 2 (5.3) | 0 (0.0) | 2 (3.3) | 0.3896 | - | - |
| Class II | 16 (42.1) | 8 (42.1) | 26 (42.6) |  |  |  |
| Class III | 20 (52.6) | 11 (57.9) | 27 (44.3) |  |  |  |
| Class IV | 0 (0.0) | 0 (0.0) | 6 (9.8) |  |  |  |
| **Medical history** |  |  |  |  |  |  |
| Hypercholesterolaemia | 3 (7.9) | 1 (5.3) | 13 (21.3) | 0.1115 | 2.6 (-13.2, 18.5) | -13.4 (-28.9, 2..1) |
| Hypertension | 28 (73.7) | 15 (79.0) | 41 (67.2) | 0.5647 | -5.3 (-32.3, 21.8) | 6.5 (14.0, 26.9) |
| Current smoker | 13 (34.2) | 5 (26.3) | 16 (26.2) | 0.6717 | 7.9 (-20.9, 36.7) | 8.0 (-12.8, 28.8) |
| Alcohol consumption | 4 (10.5) | 4 (21.1) | 15 (24.6) | 0.2240 | -10.6 (-35.2, 14.2) | -14.1 (-30.8, 2.6) |
| Current Diabetes Mellitus, (%) | 11 (29.0) | 8 (42.1) | 16 (26.2) | 0.4140 | -13.1 (-43.6, 17.3) | 2.8 (-17.6, 23) |
| Stroke | 1 (2.6) | 0 (0.0) | 1 (1.6) | 1.0000 | 2.6 (-5.1, 10.4) | 1.0 (-6, 8) |
| Atrial fibrillation, (%) | 10 (26.3) | 7 (36.8) | 13 (21.3) | 0.3890 | -10.5 (-40.3, 19.2) | 5.0 (-14.5, 24.5) |
| Chronic obstructive pulmonary disease, (%) | 4 (10.5) | 3 (15.8) | 7 (11.5) | 0.8570 | -5.3 (-28.3, 17.8) | -1.0 (-14.5, 12.6) |
| Myocardial infarction, (%) | 4 (10.5) | 5 (26.3) | 3 (4.9) | **0.0329** | -15.8 (-41.8, 10.2) | 5.6 (-7.7, 18.9) |
| Coronary artery disease, (%) | 5 (13.2) | 5 (26.3) | 4 (6.6) | 0.0613 | -13.1 (-39.6, 13.3) | 6.6 (-7.9, 21.1) |
| Prior coronary artery bypass grafting, (%) | 2 (5.3) | 3 (15.8) | 2 (3.3) | 0.1534 | -10.5 (-32.3, 11.3) | 2.0 (-8.4, 12.4) |
| Prior percutaneous coronary intervention, (%) | 2 (5.3) | 3 (15.8) | 5 (8.2) | 0.4022 | -10.5 (-32.3, 11.3) | -2.9 (-15, 9.1) |
| Prior balloon aortic Valvuloplasty, % | 0 (0.0) | 0 (0.0) | 0 (0.0) | - | - | - |
| Cerebrovascular accident, (%) | 1 (2.6) | 0 (0.0) | 0 (0.0) | 0.4831 | 2.6 (-5.1, 10.4) | 2.6 (-4.6, 9.9) |
| Porcelain aorta or hostile chest Procedural Characteristics), (%) | 0 (0.0) | 0 (0.0) | 0 (0.0) | - | - | - |
| Peripheral vascular disease, (%) | 0 (0.0) | 0 (0.0) | 0 (0.0) | - | - | - |
| Overall frailty, (%) | 7 (18.4) | 4 (21.1) | 4 (6.6) | 0.0958 | -2.7 (-27.4, 22.1) | 11.8 (-4.1, 27.8) |
| Pulmonary hypertension, (%) | 0 (0.0) | 0 (0.0) | 0 (0.0) | - | - | - |
| Permanent pacemaker, (%) | 1 (2.6) | 1 (5.3) | 6 (9.8) | 0.4827 | -2.7 (-16.5, 11.3) | -7.2 (-18.4, 4) |
| Left bundle branch block, (%) | 1 (2.6) | 0 (0.0) | 5 (8.2) | 0.3763 | 2.6 (-5.1, 10.4) | -5.6 (-16.3, 5.1) |
| Right bundle branch block, (%) | 1 (2.6) | 3 (15.8) | 3 (4.9) | 0.1785 | -13.2 (-34.3, 8) | -2.3 (-11.9, 7.3) |
| Estimated glomerular filtration rate <60 mL/min | 15/36 (41.7) | 10/16 (62.5) | 22/55 (40.0) | 0.2645 | -20.8 (-54.02, 12.35) | 1.7 (-20.7, 24.0) |
| Estimated glomerular filtration rate <30 mL/min | 3 /36 (8.3) | 6/16 (37.5) | 6/55 (10.9) | **0.0256** | (-59.06, 0.73) | (-17.10, 11.94) |
| **Echocardiography findings** |  |  |  |  |  |  |
| Aortic valve mean gradient, mm Hg | 41.6 ± 11.5 (n=37) | 37.8 ± 15.8 (n=19) | 43.3 ± 15.4 (n=59) | 0.3559 | 3.8 (-4.8, 12.4) | -1.7 (-7.3, 3.9) |
| Aortic valve peak gradient, mm Hg | 67.8 ± 17.9 (n=37) | 62.5 ± 21.9 (n=19) | 71.2 ± 23.4 (n=59) | 0.2967 | 5.3 (-6.9, 17.5) | -3.4 (-12, 5.2) |
| Aortic valve peak velocity, m/s | 4.1 ± 0.5 (n=37) | 3.9 ± 0.6 (n=19) | 4.2 ± 0.7 (n=59) | 0.3017 | 0.2 (-0.1, 0.5) | -0.1 (-0.4, 0.2) |
| Aortic valve area, cm^2^ | 0.67 ± 0.19 (n=37) | 0.75 ± 0.29 (n=19) | 0.71 ± 0.24 (n=58) | 0.4426 | -0.1 (-0.2, 0.1) | -0.04 (-0.1, 0.05) |
| Left ventricular ejection fraction, % | 59.4 ± 10.1 (n=36) | 59.3 ± 7.0 (n=19) | 56.9 ± 10.1 (n=56) | 0.4047 | 0.1 (-4.8, 5.0) | 2.5 (-1.9, 6.9) |
| Systolic pulmonary artery pressure, mm Hg | 34.9 ± 15.8 (n=35) | 35.7 ± 19.9 (n=16) | 34.3 ± 11.9 (n=43) | 0.9511 | -0.8 (-12.8, 11.2) | 0.6 (-6.0, 7.2) |
| Aortic valve EOA, cm^2^ | 0.67 ± 0.19 (n=37) | 0.75 ± 0.29 (n=19) | 0.71 ± 0.24 (n=58) | 0.4426 | -0.1 (-0.2, 0.1) | -0.04 (-0.1, 0.05) |
| **Aortic regurgitation** | n=37 | n=19 | n=58 |  |  |  |
| None/Trace | 9 (24.3) | 10 (52.6) | 15 (25.9) | 0.0921 |  |  |
| Mild | 19 (51.4) | 8 (42.1) | 36 (62.1) |  |  |  |
| Moderate | 9 (24.3) | 1 (5.3) | 7 (12.1) |  |  |  |
| Severe | 0 (0.0) | 0 (0.0) | 0 (0.0) |  |  |  |
| Moderate or higher aortic regurgitation | 9 (24.3) | 1 (5.3) | 7 (12.1) | 0.1634 | 19 (-2.0, 40.1) | 12.2 (-5.3, 31.0) |
| **Mitral regurgitation** | n=37 | n=18 | n=59 |  |  |  |
| None/Trace | 14 (37.8) | 7 (38.9) | 16 (27.1) | 0.0096 |  |  |
| Mild | 22 (59.5) | 6 (33.3) | 41 (69.5) |  |  |  |
| Moderate | 1 (2.7) | 3 (16.7) | 2 (3.4) |  |  |  |
| Severe | 0 (0.0) | 2 (11.1) | 0 (0.0) |  |  |  |
| Moderate or higher mitral regurgitation | 1 (2.7) | 5 (27.8) | 2 (3.4) | 0.0066 | -25.1 (-50.6, 0.4) | -0.7 (-8.3, 7.0) |
| **Tricuspid regurgitation** | n=37 | n=18 | n=57 |  |  |  |
| None/Trace | 13 (35.1) | 11 (61.1) | 22 (38.6) | 0.1898 |  |  |
| Mild | 21 (56.8) | 4 (22.2) | 30 (52.6) |  |  |  |
| Moderate | 2 (5.4) | 1 (5.6) | 3 (5.3) |  |  |  |
| Severe | 1 (2.7) | 2 (11.1) | 2 (3.5) |  |  |  |
| Moderate or higher tricuspid regurgitation | 3 (4.3) | 3 (16.7) | 5 (8.8) | 0.6291 | -12.4 (-32.0, 14.9) | -4.5 (-12.8, 11.5) |
| **Bicuspid valve** | n=3 | n=1 | n=6 |  |  |  |
| Type 0 | 1 (33.3) | 0 (0.0) | 0 (0.0) | 0.4583 |  |  |
| Type 1a | 1 (33.3) | 1 (100.0) | 5 (83.3) |  |  |  |
| Type 1b | 1 (33.3) | 0 (0.0) | 1 (16.7) |  |  |  |
| **Procedure** |  |  |  |  |  |  |
| **Vascular access** |  |  |  |  |  |  |
| Transfemoral approach, (%) | 38 (100.0) | 19 (100.0) | 61 (100.0) | 1.00 | 0.0 (0,0) | 0.0 (0, 0) |
| Subclavian approach, (%) | 0 | 0 | 0 |  | - | - |
| Transaortic approach, (%) | - | - | - |  |  |  |
| Predilatation | 25 (65.8) | 7 (36.8) | 33 (54.1) | 0.1142 |  |  |
| **RF, Median (Q1-Q3)** | (n=33), 2 (1 - 8) | (n=17), 3 (2 - 8) | (n=47), 5 (1-9.5) | 0.2671 |  |  |
| <6% | 23 (69.7) | 10 (58.8) | 24 (51.1) | **<0.0001** |  |  |
| 6—17% | 10 (30.3) | 6 (35.3) | 19 (40.4) |  |  |  |
| > 17% | 0 (0.0) | 1 (5.9) | 4 (8.5) |  |  |  |
| **CT findings** |  |  |  |  |  |  |
| Aortic annulus perimeter, mm | 75.8 ± 7.6 (n=38) | 77.8 ± 6.4 (n=19) | 77.5 ± 5.5 (n=61) | 0.3561 | -2.0 ( -6.03, 2.03) | -1.7 ( -4.58, 1.18) |
| Aortic annulus area, mm^2^ | 444.7 ± 89.9 (n=38) | 466.9 ± 75.3 (n=19) | 464.4 ± 66.1 (n=61) | 0.3983 | -22.2 ( -69.7, 25.3) | -19.7 ( -53.86, 14.46) |
| Small annulus (aortic annulus area ≤430 mm^2^) | 14 (36.8) | 4 (21.1) | 22 (36.1) | 0.4330 | 15.7 (-12.1, 43.6) | 0.7 (19.5, 21.1) |
| Total calcium aortic valve, mm^3^ | 1047.4 ± 648.8 (n=38) | 948.0 ± 698.2 (n=19) | 1132.0 ± 687.0 (n=61) | 0.5600 | 99.4 ( -303.27, 502.07) | -84.6 ( -362.53, 193.33) |
| **Aortic annulus characteristics** |  |  |  |  |  |  |
| Min diameters | 20.8 ± 2.3 (n=38) | 21.3 ± 2.2 (n=19) | 21.5 ± 2.0 (n=61) | 0.3114 | -0.5 ( -1.82, 0.82) | -0.7 ( -1.62, 0.22) |
| Max diameter, mm | 27.0 ± 2.6 (n=38) | 27.6 ± 2.2 (n=19) | 27.4 ± 2.1 (n=61) | 0.5495 | -0.6 ( -1.98, 0.78) | -0.4 ( -1.41, 0.61) |
| Mean diameter, mm | 23.9 ± 2.4 (n=38) | 24.4 ± 2.0 (n=19) | 24.5 ± 1.8 (n=61) | 0.3818 | -0.5 ( -1.76, 0.76) | -0.6 ( -1.52, 0.32) |
| Perimeter derived diameter, mm | 24.1 ± 2.4 (n=38) | 24.7 ± 2.1 (n=19) | 24.7 ± 1.8 (n=61) | 0.3615 | -0.6 ( -1.9, 0.7) | -0.6 ( -1.52, 0.32) |
| Annulus derived diameter, mm | 23.7 ± 2.4 (n=38) | 24.3 ± 2.0 (n=19) | 24.3 ± 1.7 (n=61) | 0.3404 | -0.6 ( -1.86, 0.66) | -0.6 ( -1.5, 0.3) |
| Perimeter, mm | 75.8 ± 7.6 (n=38) | 77.8 ± 6.4 (n=19) | 77.5 ± 5.5 (n=61) | 0.3561 | -2.0 ( -6.03, 2.03) | -1.7 ( -4.58, 1.18) |
| Area, mm^2^ | 444.7 ± 89.9 (n=38) | 466.9 ± 75.3 (n=19) | 464.4 ± 66.1 (n=61) | 0.3983 | -22.2 ( -69.7, 25.3) | -19.7 ( -53.86, 14.46) |
| **Calcification (Qualitative)** |  |  |  |  |  |  |
| AV calcification volume, mm^3^ (Quantitative) | 1047.4 ± 648.8 (n=38) | 948.0 ± 698.2 (n=19) | 1132.0 ± 687.0 (n=61) | 0.5600 | 99.4 ( -303.27, 502.07) | -84.6 ( -362.53, 193.33) |
| **AV calcification severity (qualitative)** | **n=38** | **n=19** | **n=61** |  |  |  |
| No AV calcification | 0 (0.0) | 0 (0.0) | 1 (1.6) | 0.3838 |  |  |
| Mild AV calcification | 4 (10.5) | 4 (21.1) | 5 (8.2) |  |  |  |
| Moderate AV calcification | 15 (39.5) | 8 (42.1) | 18 (29.5) |  |  |  |
| Severe AV calcification | 19 (50.0) | 7 (36.8) | 37 (60.7) |  |  |  |
| **LVOT calcification severity (qualitative)** | **n=37** | **n=18** | **n=56** |  |  |  |
| No LVOT calcification | 37 (100.0) | 18 (100.0) | 56 (100.0) | 1.0000 |  |  |
| Mild LVOT calcification | 0 (0.0) | 0 (0.0) | 0 (0.0) |  |  |  |
| Moderate LVOT calcification | 0 (0.0) | 0 (0.0) | 0 (0.0) |  |  |  |
| Severe LVOT calcification | 0 (0.0) | 0 (0.0) | 0 (0.0) |  |  |  |
| Membranous septum length | 3.8 ± 2.1 (n=14) | 4.0 ± 1.3 (n=10) | 3.9 ± 2.6 (n=31) | 0.9831 | -0.2 ( -1.77, 1.37) | -0.1 ( -1.68, 1.48) |
| Predilatation, % | 25 (65.8) | 7 (36.8) | 33 (54.1) | 0.1142 | 29.0 (-1.4, 59.3) | 11.7 (-10.0, 33.4) |
| Predilatation balloon diameter, mm | 19.8 ± 2.5 | 20.1 ± 1.5 | 21.9 ± 4.2 | 0.0563 | -0.3 ( -1.42, 0.82) | -2.1 ( -3.46, -0.74) |
| Predilation balloon vs. annulus diameters ratio | 0.9 ± 0.1 | 0.9 ± 0.1 | 0.9 ± 0.1 | 0.1542 | 0.0 ( -0.06, 0.06) | 0.0 ( -0.04, 0.04) |
| Postdilation balloon diameter, mm | 22.9 ± 4.6 | 24.7 ± 2.4 | 23.8 ± 3.0 | 0.2161 | -1.8 ( -3.75, 0.15) | -0.9 ( -2.6, 0.8) |
| Postdilation balloon vs. annulus diameters ratio | 1.0 ± 0.2 | 1.0 ± 0.1 | 1.0 ± 0.1 | 0.5099 | 0.0 ( -0.08, 0.08) | 0.0 ( -0.07, 0.07) |
| Contrast volume, ml | 171.7 ± 76.4 | 154.2 ±74.4 | 171.3 ± 89.3 | 0.7105 | 17.5 ( -26.82, 61.82) | 0.4 ( -33.77, 34.57) |
| Second valve implanted (valve-in-valve) | 0 | 0 | 1 (1.6) | - |  |  |
| **VD data** |  |  |  |  |  |  |
| **Before balloon post-dilatation:** |  |  |  |  |  |  |
| RF (before balloon post-dilation) | 12 (6 - 18.5) (n=23) | 18 (1 - 19) (n=09) | 10.5 (6 - 15) (n=26) | 0.6510 | - | - |
| RF ≤ 17 for before ballon (Yes), n(%) | 17 (73.9) | 4 (44.5) | 20 (76.9) | 0.2071 | 29.4 (-15.35, 74.29) | -3.0 (-30.19, 24.17) |
| **After balloon post-dilatation:** |  |  |  |  |  |  |
| RF (after balloon post-dilation) | 2.0 (1.0, 5.5) (n=23) | 2.0 (1.0, 3.0) (n=9) | 5.0 (1.0, 8.0) (n=26) | 0.4350 | **-** | **-** |
| RF ≤ 17 for after ballon (Yes), n(%) | 23 (100.0) | 9 (100) | 25 (96.2) | 1.00 | 0.0 (0,0) | 3.9 (-7.4, 15.1) |

Data was presented as number (Percentage), Mean ± SD, Median (Q1-Q3)

**Supplementary Table 7**: RF of final aortogram per device sizes in Myval, Sapien and Evolut THV series

| **Parameters** | **All (N=596)** | | **RF ≤0.17 (N=572)** | | **RF > 0.17 (N=24)** | |
| --- | --- | --- | --- | --- | --- | --- |
|  | **Number and proportion (%)** | **RF %** | **Number and proportion (%)** | **RF** % | **Number and proportion (%)** | **RF** % |
| **Myval** (n=289) |  |  |  |  |  |  |
| 20 | 3 (1.0) | 1 (0.5 - 2.5) | 3 (1.1) | 1 (0.5, 2.5) | 0 (0.0) | - |
| 21.5 | 11 (3.8) | 4 (1 - 7) | 11 (3.9) | 4 (1, 7) | 0 (0.0) | - |
| 23 | 61 (21.1) | 5 (1 - 8) | 60 (21.1) | 5 (1, 8) | 1 (20.0) | - |
| 24.5 | 77 (26.6) | 3 (1 - 7) | 77 (27.1) | 3 (1, 7) | 0 (0.0) | - |
| 26 | 62 (21.5) | 2 (0.3 - 6) | 60 (21.1) | 1.5 (0, 6) | 2 (40.0) | 23 (21.5 - 24.5) |
| 27.5 | 46 (15.9) | 2.5 (1 - 4.8) | 46 (16.2) | 2.5 (1, 4.8) | 0 (0.0) | - |
| 29 | 28 (9.7) | 4.5 (1.8 - 9.8) | 26 (9.2) | 4 (1.3, 7) | 2 (40.0) | 20 (20 - 20) |
| 30.5 | 1 (0.4) | - | 1 (0.35) | - | 0 (0.0) | - |
| **Sapien** (n=154) |  |  |  |  |  |  |
| 20 | 7 (4.6) | 3 (2 - 4) | 7 (4.8) | 3 (2, 4) | 0 (0.0) | - |
| 23 | 48 (31.2) | 3.5 (1 - 9.3) | 46 (31.3) | 3 (1, 8) | 2 (28.6) | 28 (27.5 - 28.5) |
| 26 | 69 (44.8) | 3 (1 - 7) | 66 (44.9) | 3 (1, 6) | 3 (42.9) | 21 (19.5 - 24.5) |
| 29 | 30 (19.5) | 1 (0 - 4.8) | 28 (19.1) | 1 (0, 4) | 2 (28.6) | 23.5 (21.3 - 25.8) |
| **Evolut** (n=153) |  |  |  |  |  |  |
| 23 | - | - | - | - | - | - |
| 26 | 32 (20.9) | 8 (1.5 - 11.3) | 30 (21.3) | 7 (0.5, 10.8) | 2 (16.7) | 20.5 (19.3 - 21.8) |
| 29 | 88 (57.5) | 4 (1 - 9) | 80 (56.7) | 4 (1, 8) | 8 (66.7) | 22.5 (18 - 26) |
| 34 | 33 (21.6) | 6 (2 - 9) | 31 (22.0) | 5 (1.5, 9) | 2 (16.7) | 28.5 (27.8 - 29.3) |

Data was presented as number (Percentage), Median (Q1-Q3)
